# Sulfide oxidation by members of the Sulfolobales

**DOI:** 10.1093/pnasnexus/pgae201

**Published:** 2024-05-23

**Authors:** Maria C Fernandes-Martins, Daniel R Colman, Eric S Boyd

**Affiliations:** Department of Microbiology and Cell Biology, Montana State University, Bozeman, MT 59717, USA; Department of Microbiology and Cell Biology, Montana State University, Bozeman, MT 59717, USA; Department of Microbiology and Cell Biology, Montana State University, Bozeman, MT 59717, USA

**Keywords:** thermophile, acidophile, archaea, chemoautotrophy, Sulfolobus

## Abstract

The oxidation of sulfur compounds drives the acidification of geothermal waters. At high temperatures (>80°C) and in acidic conditions (pH <6.0), oxidation of sulfide has historically been considered an abiotic process that generates elemental sulfur (S^0^) that, in turn, is oxidized by thermoacidophiles of the model archaeal order Sulfolobales to generate sulfuric acid (i.e. sulfate and protons). Here, we describe five new aerobic and autotrophic strains of Sulfolobales comprising two species that were isolated from acidic hot springs in Yellowstone National Park (YNP) and that can use sulfide as an electron donor. These strains significantly accelerated the rate and extent of sulfide oxidation to sulfate relative to abiotic controls, concomitant with production of cells. Yields of sulfide-grown cultures were ∼2-fold greater than those of S^0^-grown cultures, consistent with thermodynamic calculations indicating more available energy in the former condition than the latter. Homologs of sulfide:quinone oxidoreductase (Sqr) were identified in nearly all Sulfolobales genomes from YNP metagenomes as well as those from other reference Sulfolobales, suggesting a widespread ability to accelerate sulfide oxidation. These observations expand the role of Sulfolobales in the oxidative sulfur cycle, the geobiological feedbacks that drive the formation of acidic hot springs, and landscape evolution.

**Significance Statement**

The oxygen-dependent oxidation of elemental sulfur by members of the model archaeal order Sulfolobales is thought to acidify hydrothermal waters. However, the primary source of sulfur in most hydrothermal systems is sulfide, which is widely thought to spontaneously oxidize in the presence of oxygen. Here, we show that sulfide is stable in the presence of oxygen at acidic pH and that aerobic Sulfolobales significantly accelerate its oxidation and couple this to cell and acid production. Growth kinetics were significantly enhanced in cultures provided with sulfide relative to elemental sulfur, suggesting sulfide as the preferred electron donor. These results expand the role of Sulfolobales in the geobiological feedbacks that modulated the coevolution of thermoacidophiles and their acidic habitats.

## Introduction

Volcanic hot springs exhibit a bimodal distribution in their pH, reflecting the prevalence of two types of spring waters: acid-sulfate (pH 2–4) and neutral-bicarbonate (pH 7–9), respectively (1). Formation of acid-sulfate waters is initiated by injection of magmatic sulfur dioxide (SO_2_) into deep hydrothermal fluids and its subsequent disproportionation as those fluids cool (<400°C) to form either (i) sulfuric acid (HSO_4_^−^), elemental sulfur (S^0^), and protons (H^+^) or (ii) HSO_4_^−^, sulfide (H_2_S/HS^−^), and H^+^ (Eqs. 1 and 2, respectively) (2). Reaction 2 is expected to prevail in volcanic systems with lower sulfur concentrations and lower temperatures (3), such as YNP (4). During their ascent to the surface, fluids can undergo the process of decompressional boiling, which allows volatiles (e.g. hydrogen sulfide or H_2_S) to partition into the gas phase (4, 5) and that leaves a volatile poor liquid phase that forms neutral-bicarbonate (pH 7–9) hot spring waters when it surfaces. Low density volatiles can continue to ascend to the surface where they can condense with oxygen (O_2_)-rich near surface groundwaters (1, 6). Under such conditions, sulfide can react with O_2_ to form thiosulfate (S_2_O_3_^2−^) (Eq. 3) that, in acidic waters (< 6.0), rapidly disproportionates to form S^0^ and bisulfite (HSO_3_^−^) (Eq. 4; (7)). HSO_3_^−^ is also unstable in acidic and oxygenated waters and oxidizes rapidly to form SO_4_^2−^ (Eq. 5).


(1) $$3SO_{2}+3H_{2}O\to 2\mathrm{HS}O_{4}^{-}+S^{0}+2H^{+}$$

(2) $$4SO_{2}+4H_{2}O\to 3\mathrm{HS}O_{4}^{-}+H_{2}S+3H^{+}$$

(3) $$2{\mathrm{HS}}^{-}+2O_{2}\to S_{2}O_{3}^{2-}+H_{2}O$$

(4) $$S_{2}O_{3}^{2-}+H^{+}\to S^{0}+\mathrm{HS}O_{3}^{-}$$

(5) $$\mathrm{HS}O_{3}^{-}+\frac{1}{2}O_{2}\to SO_{4}^{2-}+H^{+}$$

(6) $$S^{0}+3/2O_{2}+H_{2}O\to \mathrm{HS}O_{4}^{-}+H^{+}.$$

At temperatures <100°C, S^0^ is stable and thus represents an abundant electron donor and acceptor for microorganisms in hot spring environments (4, 7). Reactions 1–5 are generally considered abiotic and do not contribute to net production of acidity (7). However, the oxidation of S^0^ is considered biologically mediated, a reaction that is thought to be responsible for the acidification of hot springs (4, 7–11).

At temperatures above 80°C, the dominant organisms in acidic hot springs are generally members of the archaeal order Sulfolobales (12–15). While aerobic S^0^-oxidation is generally the metabolism associated foremost with members of the Sulfolobales (8, 9, 16, 17), the group is metabolically diverse and reports of heterotrophic or autotrophic, aerobic or anaerobic, and lithotrophic growth are widespread among cultivars (16–19). For example, members of the Sulfolobales have been shown to grow via oxidation of ferrous iron (Fe(II)), hydrogen (H_2_), and pyrite (FeS_2_), via reduction of ferric iron (Fe(III)) and S^0^, and via S^0^-disproportionation, among other oxidation-reduction reactions. Nevertheless, aerobic S^0^-oxidation was long thought to be a unifying feature of Sulfolobales until more recent reports of Sulfolobales strains that are either inhibited by S^0^ (*Sulfodiicoccus acidiphilus*) (20) or that are strict anaerobes (*Stygiolobus azoricus* (21)).

S^0^-oxidation in Sulfolobales is thought to involve the cytoplasmic enzyme sulfur oxidoreductase:reductase (Sor) that catalyzes the O_2_-dependent disproportionation of S^0^ to yield sulfide, HSO_3_^−^, and S_2_O_3_^2−^, a reaction that by itself is not energy conserving (19, 22–25). Sulfide is then oxidized by membrane-associated sulfide:quinone oxidoreductase (Sqr) that conserves energy by coupling oxidation to the reduction of quinone in a membrane-associated electron transport chain (26), while HSO_3_^−^ and S_2_O_3_^2−^ are processed downstream through additional energy conserving reactions (19, 27, 28). As such, any Sulfolobales that can oxidize S^0^ via Sor should also be able to oxidize sulfide. Indeed, all Sulfolobales that encode Sor also encode Sqr (19, 22). Further, those members of the Sulfolobales that do not encode Sor encode Sqr and a heterodisulfide reductase (Hdr) complex (16, 19, 22), and the latter has been suggested to be an alternative and essential pathway for sulfur oxidation in other thermophiles, including model acidophilic bacteria (29–32), as well as in the Sulfolobales strain *Metallosphaera cuprina* (32). Together, these observations raise the question as to whether Sulfolobales can catalyze sulfide oxidation and couple this redox reaction to biomass production as an alternative or preferred strategy over S^0^-oxidation.

In potential support of sulfide oxidation by Sulfolobales, a Sqr purified from membranes of *Acidianus ambivalens* linked sulfide oxidation to O_2_-reduction (26). Further, Sqr purified from membranes of the archaeon *Caldivirga manquilingensis* was shown to have the same catalytic properties of linking sulfide oxidation to the reduction of quinone (33). In addition, studies of high temperature hot spring communities have interpreted the presence of Sqr homologs in reconstructed Sulfolobales genomes as evidence that they are involved in sulfide oxidation (29, 34, 35). However, despite 50+ years of study, oxidation of sulfide by members of the Sulfolobales (or more broadly among Archaea) remains an open question (16, 19) since the few papers that have reported oxidation of sulfide by Sulfolobales either did not show the supporting data (36–38) or provided debatable results (39–41), as discussed more below. It is also possible that the prevailing notion that sulfide is unstable in the presence of O_2_ at high temperature and in acidic conditions (4, 7, 8) may have limited research into whether Sulfolobales can accelerate this oxidative process.

To begin to reconcile these observations, we investigated abiotic and biotic reactions involved in sulfide oxidation at high temperature and acidic pH. We probed the kinetics of abiotic sulfide oxidation with O_2_ at varying pH, temperature, and O_2_ concentration to identify conditions where microorganisms could possibly accelerate the kinetics of the reaction and conserve energy from the reaction. This information was then used to guide the enrichment of sulfide-oxidizing organisms from three hot springs in Yellowstone National Park, Wyoming, USA: Cinder Pool, “Realgar Pool”, and “Red Bubbler”. We describe the ability of four closely related strains of *Stygiolobus* (Sulfolobales) and a new genus (Sulfolobales) to accelerate the O_2_-dependent oxidation of sulfide, generating acidity through SO_4_^2−^ and H^+^ production. These data expand the role of Sulfolobales in the oxidative sulfur cycle to include sulfide oxidation and further underscore their role in the acidification of hot springs waters.

## Materials and methods

### Kinetics of abiotic sulfide oxidation

Abiotic sulfide oxidation assays were conducted in laboratory defined conditions at 80°C and at pH 3.0 or 7.0 and in the presence of varying O_2_ concentrations in the headspace (0%, 1%, and 21% vol./vol.). Ten milliliters of Milli-Q water were distributed in acid-washed 24 mL serum bottles and vials were sealed with grey butyl rubber stoppers. Following autoclave sterilization, vials were purged for 20 min. with N_2_ passed over heated (350°C) and H_2_-reduced copper shavings. Triplicate vials for each condition were prepared for each time point. An aliquot of anoxic and filter-sterilized (0.22 µm) sodium citrate solution (prepared to final pH of 3.0) or Tris-HCl (prepared to a final pH of 7.0) was added to each serum bottle to a final concentration of 200 µM or 1 mM, respectively. The N_2_ headspace of vials was left as is (0% O_2_ condition) or was replaced by air at a specific volume to achieve the final concentration of 1% vol./vol. O_2_, or completely replaced by air to achieve the final concentration of 21% vol./vol O_2_. Finally, an aliquot of an anoxic and filter-sterilized (0.22 µm) solution of sodium sulfide (Na_2_S) was added to each serum bottle to a final concentration of 100 µM. The experiments were run for 15 h and the concentration of dissolved total sulfide (H_2_S/HS^−^/S^2−^) was determined every 3 h via the methylene blue reduction assay (42), and this was converted to total sulfide using Henry's law as previously described (43).

A second set of abiotic sulfide oxidation kinetic assays was conducted to investigate the reactivity of sulfide with ferric iron ions [added as ferric sulfate (Fe_2_SO_4_)_3_]. These experiments were conducted at pH 2.6 and at 80°C, and the vials were prepared as described above (without addition of headspace O_2_) to mimic previously described experiments (39). Briefly, Na_2_S was added to a final concentration of 8 mM in triplicate 70 mL serum bottles with 30 mL of Milli-Q containing 25 mM (Fe_2_SO_4_)_3_. A triplicate set of serum bottles that did not contain (Fe_2_SO_4_)_3_ was used as a control. All reactors were buffered with nine mM of an anoxic and filter-sterilized (0.22 µm) sodium citrate solution (prepared to final pH of 2.6). Aqueous sulfide concentrations were measured and converted to total sulfide as described above, and production of ferrous iron was determined via the Ferrozine assay (44).

### Sample collection and field geochemical assays

In 2018, ∼1 g of sediment from 14 acidic hot springs (Table S4) in YNP were aseptically sampled, placed in sterile 15 mL falcon tubes, frozen on dry ice in the field and stored in a −80°C freezer back in the laboratory until used in DNA extractions. Subsamples of hot spring water for enrichment and cultivation were collected on 2020 August 28th, in autoclaved 70 mL glass serum vials from Cinder Pool (CP; pH 2.6, 87.8°C; 44.732444 N, 110.709779 W) and from the surface of “Realgar Pool” (RP; pH 3.9, T 85.8°C; 44.73558 N, 110.70705 W), both of which are located at Norris Geyser Basin, YNP. On 2021 June 3rd, samples of hot spring water were again collected from CP but from 9 and 21 m below the surface for cultivation and activity assays. A description of the approach to collect samples from depth at CP is discussed elsewhere (29). On 2023 June 13th, samples for cultivation were collected from the surface water of “Red Bubbler” (RB; pH 3.0, T 90°C; 44.72650 N, 110.70900 W), also located at Norris Geyser Basin. Sterile vials for water samples for cultivation were completely filled and capped with sterile butyl rubber stoppers equipped with needles to exclude headspace. The needles were then removed, the bottles capped, wrapped in aluminum foil to exclude light, and kept at room temperature (∼20°C) during transport to the laboratory prior to inoculating enrichment medium. Surface water from each hot spring for use as an additive to growth medium (described below) was also collected from each spring and filtered (0.22 µm) in the field into autoclaved polypropylene bottles that were then stored on ice during transport back to the laboratory, followed by storage at 4°C until their use.

The temperature, conductivity, and pH of hot spring waters were measured in the field with temperature-compensated probes (model YSI EC300, YSI Inc., Yellow Springs, OH, USA or model WTW 3100, WTW Weilheim, Germany). Subsamples of waters for ion chromatography (IC), inductively coupled plasma mass spectrometry (ICP-MS), and ICP-Optical Emission Spectroscopy (ICP-OES) were filtered in the field (0.22 µm) and stored in polypropylene vials capped with no headspace. Samples for ICP-MS/ICP-OES were acidified with trace metal grade nitric acid to a final concentration of 1% vol./vol. Samples of culture medium or supernatant following growth were filtered (0.22 µm) prior to acidification for ICP-MS analyses. IC, ICP-MS, and ICP-OES analyses of hot spring waters were conducted at the Montana Bureau of Mines and Geology Analytical Laboratory.

### Enrichment and isolation

Enrichment medium was prepared by combining 80% base salt medium with 20% filtered (0.22 µm) water from either CP or RP. Base salt medium comprised CaCl_2_ • 2H_2_O (0.33 g L^−1^), NH_4_Cl (0.33 g L^−1^), KCl (0.33 g L^−1^), MgCl_2_ • 6H_2_O (0.33 g L^−1^), and KH_2_PO_4_ (0.33 g L^−1^), as previously described (43). The salts were added to Milli-Q water and the pH was adjusted to 2.6 (CP) or 4.0 (RP) using 1N HCl. Twenty-seven milliliters of base salt/filtered spring water medium was dispensed into 70 mL serum bottles that were then sealed with grey butyl rubber stoppers. Following autoclave sterilization, vials were purged for 20 min. with N_2_ passed over heated (350°C) and H_2_-reduced copper shavings. Next, the headspace was purged with carbon dioxide (CO_2_) for 5 min. and vials were placed in an 85°C incubator. After 2 h of incubation, the headspace was equilibrated to atmospheric pressure, followed by addition of anoxic and filter-sterilized (0.22 µm) solutions of Wolfe's vitamins (45) and SL-10 metals (46) to a final concentration of 1 mL L^−1^ each. Oxygen (O_2_) (as air) was added to the headspace to a final concentration of 1.5% vol./vol. An anoxic and filter-sterilized (0.22 µm) sodium citrate buffer solution, prepared to a final pH of 2.6 (CP) or 4.0 (RP), was added to serum bottles to a final concentration of 200 µM and an anoxic and filter-sterilized (0.22 µm) solution of Na_2_S was added to each serum bottle to a final concentration of 100 µM. Three milliliters of a sediment/spring water slurry were added to each respective vial as inoculum and vials were incubated at 85°C. For elemental sulfur (S^0^) enrichment conditions, serum bottles were prepared as described above with the exception that baked (100°C, 120 min) S^0^ was added to a final concentration of ∼450 µM (0.014 g L^−1^). To better understand why the Sulfolobales strains grow better with filter-sterilized hot spring water additions, cultures were grown under S^0^ oxidizing conditions and compared using 100% base salt medium vs. 80% synthetic base salt medium with 20% sterile hot spring water. Triplicate cultures were prepared for each condition for the initial time point (TI) and for the final time point (TF) (*n* = 12). Following incubation, culture medium was filter-sterilized (0.22 µm), and the filtrate prepared for ICP-MS analyses, as described above. ICP-MS was conducted at the Montana Bureau of Mines and Geology Analytical Laboratory.

### Monitoring of growth and activity in enrichments

Enrichment progress was evaluated every 5 h by monitoring the concentrations of dissolved sulfide [primarily H_2_S at pH 2.6 and 4.0 (47)], SO_4_^2−^, and cells. The concentration of aqueous sulfide was determined via the methylene blue reduction assay (42), and this was converted to total sulfide in each serum bottle reactor using Henry's law as previously described (43). The concentration of SO_4_^2−^ was determined via a barium chloride turbidity assay (48). Quantification of sulfite (HSO_3_^−^) and thiosulfate (S_2_O_3_^2−^) were probed in spent culture media with assays based on the reduction of fuchsin (49, 50) and ion-chromatography (IC), respectively. The concentration of cells in enrichments was determined by removing 0.5 mL aliquots of culture, mixing with 0.1 µL of a 2 mg mL^−1^ 4′,6-diamidino-2-phenylindole (DAPI) stock, and incubating the mixture in the dark for ∼30 min. Cells were collected onto black polycarbonate filters with 0.22 µm pores (Millipore Sigma, Billerica, MA, USA) and were enumerated with an Evos fluorescence microscope (Thermo Fisher Scientific, Waltham, MA, USA). Cultures were transferred until a single morphotype was observed and were maintained by weekly (10% vol./vol.) transfers of log or late log phase cells into fresh growth medium.

### DNA extraction, (meta)genomic sequencing, assembly, and genome binning

Genomic DNA was extracted from sediment samples (∼0.5 g) from the 14 hot springs (Table S4) and from enrichment culture biomass using the FastDNA Sping Kit for Soil (MP Biomedicals, Irvine, CA, USA) following the manufacturer's instructions and was quantified fluorometrically via the high sensitivity Qubit assay (Thermo Fisher Scientific). Biomass from cultivation vials for DNA extractions was concentrated via centrifugation (14,000 × *g*, 20 min, 4°C) from 300 mL of enrichment culture that was confirmed via microscopy to have a single morphotype. Genomic DNA from the 14 hot spring sediment samples was subjected to paired-end sequencing (2 × 151 bp) with the Illumina NovaSeq platform at the UW Madison Next-Generation Sequencing Center. Genomic DNA from enrichment cultures was sequenced via the Illumina NovaSeq platform at Microbial Whole Genome Sequencing (MiGs; Pittsburgh, PA, USA). Read processing, genome assembly, and binning of contigs into metagenome-assembled-genomes (MAGs) was performed as previously described (51) using the MetaWRAP pipeline (52). MAGs recovered from hot spring metagenomes are deposited at the National Center for Biotechnology Information (NCBI) database (53) under BioProject PRJNA1019763. The partial genome sequences of the isolates are deposited in the NCBI database under BioProject PRJNA1019763, together with their translated protein content, with the exception of the genome of Sulfolobales RB85. Protein homologs encoded in the Sulfolobales RB85 genome can be found in Table S8.

### Phylogenetic and genomic characterization

Marker genes (*n* = 30) for Archaea were identified, aligned, and concatenated using Markerfinder (https://github.com/faylward/markerfinder#markerfinder) for 31 reference genomes or MAGs (four representative of outgroups for the Sulfolobales order, 21 type strain of the Sulfolobales order, and the five isolated strains). The alignment block was subjected to phylogenetic reconstruction using IQ-Tree (v.1.6.11) (54) specifying the LG model and 1,000 “ultrafast” bootstrap replicates, as previously described (51). Open reading frames and encoded proteins were identified with PROKKA (v.1.14.5) (55), and the protein annotation for each MAG was then submitted to METABOLIC v 4.0 (56) to identify key genes for CO_2_ fixation and dissimilatory sulfur metabolism. This included 4-hydroxybutyryl-CoA dehydratase (Abfd) for CO_2_ fixation and sulfide quinone oxidoreductase (Sqr), sulfur oxygenase:reductase (Sor), heterodisulfide reductase (HdrAB1B2C1C2), and sulfur/polysulfide reductase (SreABC) for dissimilatory sulfur metabolism (Table S5). In addition, homologs of Sqr were identified among 51 hot spring metagenomes from our in-house hot spring database (Tables S4 and S6) using METABOLIC v 4.0 (56). The homologs for sulfite:acceptor oxidoreductase (Suox), thiosulfate:quinone oxidoreductase (Tqo), and tetrathionate hydrolase (TetH) were identified with query proteins from *Acidianus ambivalens* and the Basic Local Alignment Search Tool (BLASTp) (57). Parameters used to search for homologs were an E value cutoff of 1.0e^−50^, amino acid identify of >50%, and >60% coverage of the query sequence (51). BLASTp was also used to determine the percent identify between the biochemically characterized Sqr from *A. ambivalens* and Sqr homologs identified in the genomes of isolates. Homologs that were not in the publicly available assemblies or were encoded on unbinned contigs are available in Table S9.

## Results

### Kinetics of abiotic sulfide oxidation

**Fig. 1.**
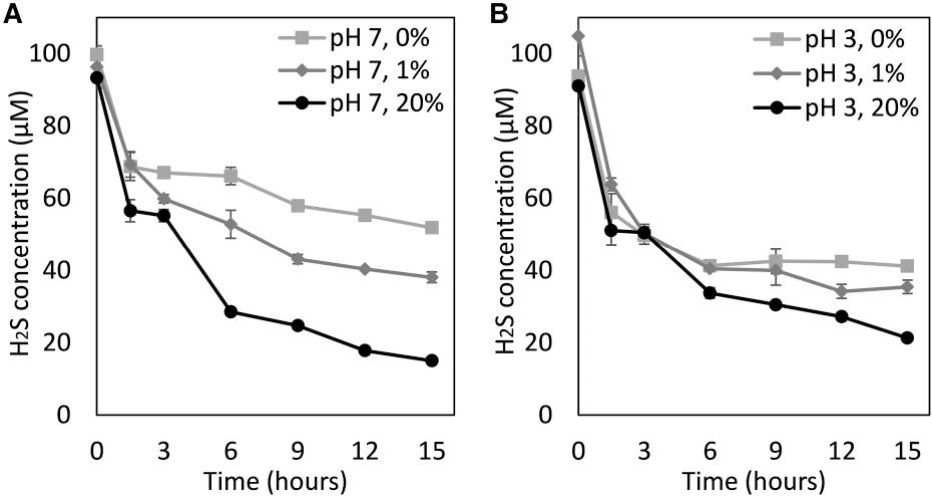
Kinetics of equilibration and abiotic oxidation of sulfide (added as Na_2_S). Experiments were conducted in bicarbonate-buffered (1 mM; pH 7.0) Milli-Q water (A) or citric acid-buffered (0.2 mM; pH 3.0) Milli-Q water (B) in reactors with headspace O_2_ concentrations of 0%, 1%, and 21% vol./vol. Reactors were incubated at 80°C and sub-samples for measurement of aqueous sulfide were taken every 3 h over a 15 h period. The average and standard deviation of triplicate measurements is shown; in some time series measurements, the standard deviation is not visible. Rates of total sulfide oxidation, which account for both aqueous and gas phase sulfide via Henry's law calculations, are presented in the text.

The kinetics of abiotic sulfide oxidation were determined at 80°C in Milli-Q water buffered at a pH of 3.0 or a pH of 7.0 and in the presence of 0%, 1%, and 21% O_2_ (vol./vol.) (Fig. 1). The concentration of aqueous sulfide was quantified every 3 h for a period of 15 h. The decrease in aqueous sulfide in the 0% O_2_ condition conducted at pH 3.0 and pH 7.0 indicated that it quickly equilibrated with the gas phase, since no other oxidants were available (Fig. 1). The equilibration period occurred within the first 6 h in the pH 3.0 assays whereas it occurred throughout the duration of the experiment in assays conducted at pH 7.0. Therefore, the difference observed in the depletion of sulfide between the 0% O_2_ (vol./vol.) assays (equilibration only) when compared to those conducted in the presence of 1 and 21% O_2_ (vol./vol.) assays (equilibration + oxidation) can be used to isolate the amount of sulfide that was oxidized abiotically by O_2_.

In assays conducted at pH 7.0 and in the presence of 1% and 21% O_2_ vol./vol, the concentration of sulfide decreased to below the equilibrium concentrations (0% O_2_ vol./vol.) within the first 3 h of incubation (Fig. 1A). In comparison, assays conducted at pH 3.0 in the presence of 1% and 21% O_2_ showed minimal decreases below that of the equilibrium concentration (0% O_2_ vol./vol.) after 3 h (Fig. 1B). The rate of abiotic oxidation of sulfide at 1% and 21% vol./vol. O_2_ was calculated between 3 and 15 h by transforming the measured aqueous sulfide concentration (µM) to total sulfide in µmoles (aqueous and gas phase). The rate of abiotic oxidation in assays provided with 1% vol./vol. O_2_ was 0.03 ± 0.005 and 0.04 ± 0.005 µmols h^−1^ at pH 3.0 and pH 7.0, respectively. Assays provided with 21% vol./vol. O_2_ exhibited an abiotic sulfide oxidation rate of 0.06 ± 0.006 and 0.08 ± 0.002 µmols h^−1^ at pH 3.0 and pH 7.0, respectively.

A second experiment was conducted to investigate the kinetics of abiotic oxidation of sulfide by ferric ions [Fe(III) added as (Fe_2_SO_4_)_3_] under anoxic conditions. Abiotic experiments were established to mimic the conditions used in Plumb et al. (2007). Anoxic citric acid-buffered medium containing 25 mM (Fe_2_SO_4_)_3_ was reacted with 8 mM of sodium sulfide (Na_2_S) at 80°C and at a pH 2.6. A near instantaneous reaction occurred (Video S1) as evinced by the immediate formation of black suspended particles that quickly transformed into a white flocculant material that then precipitated. XRD analysis of the precipitated flocs showed a 100% match with S^0^ (Fig. S7). These reactions resulted in an immediate consumption of all added sulfide and the concomitant production of ∼12.5 mM Fe(II), which nearly accounts for the 8 mM sulfide if all of it was converted to S^0^ (release of 16 mM electrons). These observations are consistent with the results presented by Plumb et al. (2017) wherein the concentration of Fe(II) at the start of the experiment was ∼9 mM, despite those authors having not added Fe(II) but rather having added Fe(III).

### Isolation of sulfide-oxidizing Sulfolobales strains

Water from the surface (0 m) of two YNP hot springs, RP (pH 4.0) and CP (pH 2.6; Tables S1 and S2, Fig. S1), was used as inoculum for enrichment of aerobic, autotrophic sulfide-oxidizing microorganisms. Additional sampling campaigns were conducted to collect water from 9 and 21 m depth (pH 2.6) from CP and from the surface of an additional hot spring, RB (pH 3.0), for use as inoculum for enrichment using the same strategy as above. Initial enrichments failed to produce cells unless a small amount of filter sterilized (0.22 µm) and autoclaved spring water was added to the microcosms. ICP-MS was conducted on culture supernatants to identify elements present in hot spring water that might stimulate cell growth. No trends in metal concentration pre- and post-growth were identified with the exception of iron (Fe) that decreased in concentration (Table S7). However, a decrease in Fe was observed in cultures grown with both 100% base salt medium and the 4:1 ratio of base salt medium to filtered and autoclaved hot spring water, suggesting that this was unlikely to be what was stimulating growth. Rather, it is possible that an element not part of those analyzed via ICP-MS or an organic substrate present in spring water may be what is stimulating growth. Subsequent enrichments and enrichment transfers therefore contained a 4:1 ratio of base salt medium to filtered and autoclaved hot spring water. Repeated passage of enrichments from 0, 9, and 21 m depth at CP, in addition to those enrichments from surface (0 m) from RP and RB resulted in single morphotypes (cocci) for each of the five samples. SEM images of the YNP strain CP85–0 m reveal 0.8 to 1.2 µm diameter coccoid cells (Fig. S3), consistent with results from fluorescent microscopy and with the morphology of Sulfolobales in general (8, 19, 58).

### Genomic sequencing, characterization, and phylogenomic analyses

Genomic DNA was extracted and sequenced from each of the five enrichments. Assembly resulted in a single genome for each isolate, adding support to microscopic analyses indicating that each culture hosted a single morphotype. A phylogenomic reconstruction of the five isolate genomes in the context of 26 Sulfolobales metagenome-assembled genomes (MAGs) recovered from 14 acidic YNP hot springs, and 26 reference genomes (21 type strains of the Sulfolobales order and five outgroup genomes: *Desulfuroccocus amylolyticus*, *Desulfuroccocus mucosus*, *Thermogladius calderae*, *Thermosphaera aggregans*) was constructed (Fig. S2). The isolates from RP and CP formed a well-supported clade with *Stygiolobus azoricus*, the type strain of this genus. The genomes of cultivars from depth at CP (CP85–9 m and CP85–21 m) formed a well-supported and distinct lineage from those of the CP and RP surface strains (CP85–0 m and RP85–0 m). The four YNP *Stygiolobus* genomes share 94.0% average nucleotide identity (ANI) with the type strain, *S. azoricus*, and are highly similar to each other (pairwise ANIs of 98.4 to 99.0%) (Table S3). The three *Stygiolobus* genomes (CP85–0 m, CP85–9 m, and CP85–21 m) from CP cultivars all had estimated genome sizes of 2.3 Mbp while the *Stygiolobus* RP85–0 m genome had a genome size of 1.9 Mbp. All genomes were estimated to be >87% complete. Additional details of cultivar genomes, including completeness, contamination, and N50 of contigs, are reported in Table S3.

The genome of the RB85–0 m cultivar formed a well-supported and distinct branch relative to the *Sulfurisphaera* and *Sulfolobus* clades, with an ANI of 74.9, 76.2, and 73.0% to *Sulfurisphaera tokodaii*, *Sulfurisphaera ohwakuensis,* and *Sulfolobus acidocaldarius,* respectively. The average amino acid identity (AAI) was 66.8% to both *Sulfurisphaera* species and 63.0% to *Sulfolobus acidocaldarius*. Similarly, the 16S rRNA gene from the RB85–0 m genome shared 95% sequence identity with *S. tokodaii* and *S. ohwakuensis*. This suggests that RB85–0 m likely represents a separate and new genus or species within the Sulfolobales. The Sulfolobales genome from RB had an estimated genome size of 2.4 Mbp and was estimated to be 99.4% complete (Table S3).

To compare metabolic pathways encoded by the RB85–0 m genome, the YNP *Stygiolobus* genomes (*n* = 4), reference Sulfolobales genomes (*n* = 21) and Sulfolobales MAGs recovered from metagenomes from 14 acidic hot springs in YNP (*n* = 26), the presence of key homologs related to CO_2_-fixation (Abfd), dissimilatory sulfur metabolism (Sqr, Sor, Hrd, Sre), and to O_2_ reduction (Cox) were mapped onto the phylogenetic tree (Fig. S2). The reference Sulfolobales genomes, the Sulfolobales MAGs recovered from YNP, and all five isolate YNP Sulfolobales genomes (CP85–0 m, RP85–0 m, CP85–9 m, CP85–21 m, and RB85–0 m) encoded similar metabolic potentials with differences observed mainly in the distribution of homologs of genes encoding two enzyme complexes (Sor and SreABC). All reference Sulfolobales genomes (with the exception of *Sulfodiicoccus acidiphilus*), all Sulfolobales MAGs recovered from YNP (with the exception of *Saccharolobus caldissimus* RB8), and the five new YNP Sulfolobales genomes encoded the ability to fix CO_2_ through the 3-hydroxypropionate/4-hydroxybutyrate pathway (3HP/4HB), as indicated by presence of the protein homolog diagnostic for this pathway, Abfd. All Sulfolobales analyzed (reference, YNP recovered MAGs, and isolates) encoded the ability to reduce O_2_, as indicated by the presence of Cox. All reference Sulfolobales genomes, with the exception of *Sulfolobus acidocaldarius*, and all Sulfolobales MAGs recovered from YNP springs encoded homologs of Sqr. Purified Sqr from *A. ambivalens* (Sulfolobales), which commonly inhabit acidic hot springs, was shown to catalyze the oxidation of sulfide coupled to reduction of quinone, with polysulfide as the putative product of oxidation (26). These catalytic properties were also subsequently shown in purified Sqr from *C. manquilingensis* (Thermoproteales), that are also common inhabitants of acidic hot springs (33). Reference genomes such as Sulfolobales Acd1 (Yellowstone group), *Sulfolobus acidocaldarius, Stygiolobus azoricus, Saccharolobus caldissimus, Saccharolobus solfataricus, Saccharolobus islandicus, Saccharolobus shibatae, Sulfodiicoccus acidiphilus, Metallosphaera yellowstonensis, Metallosphaera hakonensis,* and *Metallosphaera cuprina* lacked homologs of Sor but encoded homologs of the Hdr complex (HdrAB1B2C1C2), which is suggested to be important for S^0^ oxidation in other thermophiles (30–32). Only nine reference genomes (*Sulfurisphaera ohwakuensis, Stygiolobus azoricus, Saccharolobus caldissimus, Saccharolobus solfataricus, Saccharolobus islandicus, Metallosphaera yellowstonensi, Acidianus brierleyi*, *Acidianus sulfidivorans,* and *Acidianus manzaensis*) and only two of the recovered YNP MAGs (Sulfolobales Acd1 Yellowstone AL7 and OHSP4) encoded homologs of SreABC, indicating an ability to respire S^0^ via this pathway. All four *Stygiolobus* genomes (CP85–0 m, RP85–0 m, CP85–9 m, and CP85–21 m) encoded homologs of Sqr allowing for sulfide oxidation and Sor or the Hdr complex allowing for S^0^ oxidation. Sulfolobales RB85–0 m encoded only Sqr and the Hdr complex (Fig. S2 and Table S5). All the five Sqr present in the isolated strains exhibited >80% sequence identities (100% sequence coverage) to Sqr from *Acidianus ambivalens* (26), suggesting that their functions are comparable.

### Kinetics of sulfide oxidation and cell production

**Fig. 2.**
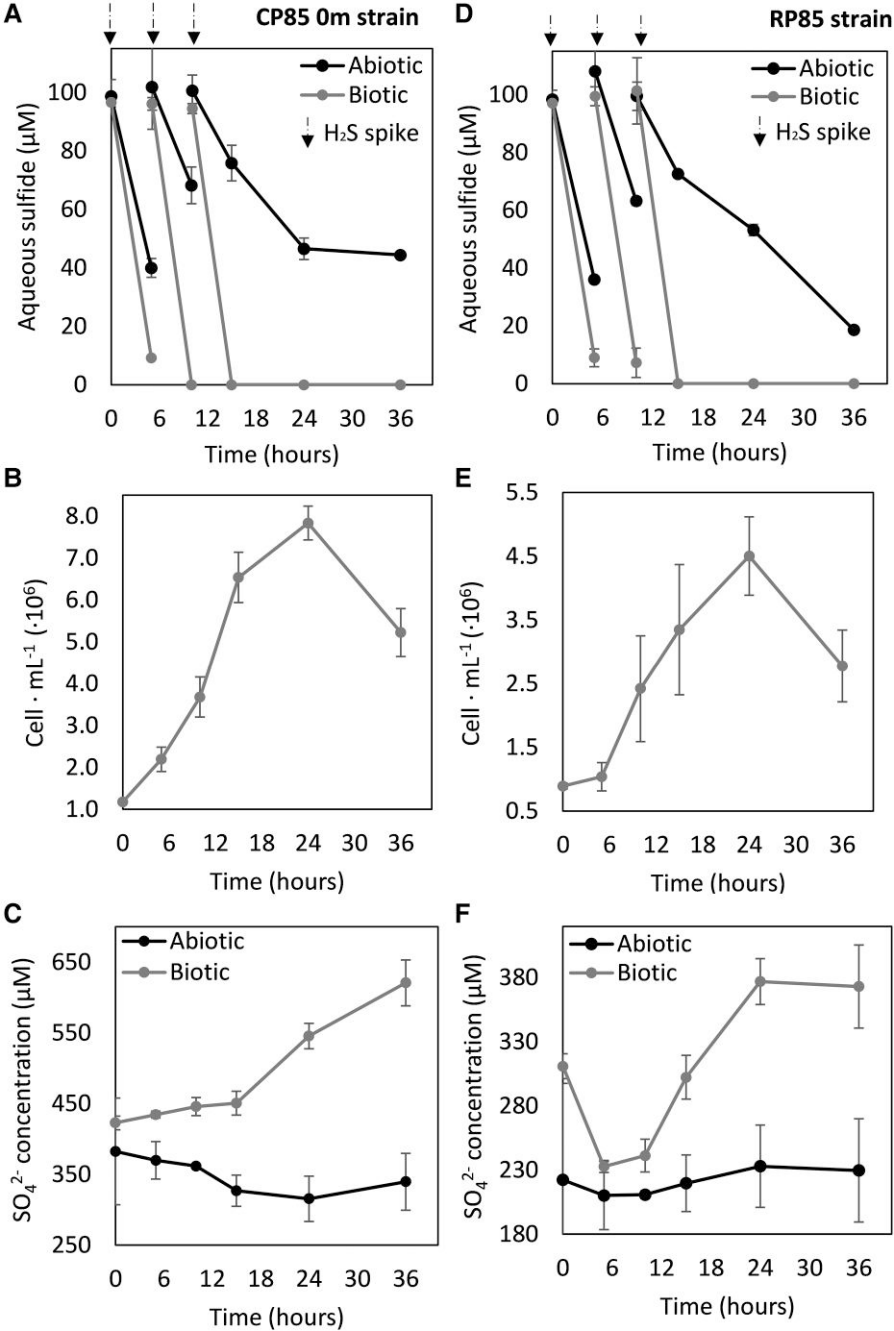
Depletion of aqueous sulfide, production of cells, and production of sulfate (SO_4_^2−^) in Sulfolobales isolates recovered in this study. Data are presented for cultures of *Stygiolobus* strain CP85–0 m grown in base salts medium with a pH of 2.6 (A–C) and in cultures of *Stygiolobus* strain RP85–0 m in base salts medium with a pH 4.0 (D–F) when incubated at 85°C. Data for abiotic controls are included where appropriate. Oxygen (1.5% headspace vol./vol.) was the electron acceptor and carbon dioxide (92% vol./vol.) was the carbon source. Black arrows depict additions of sulfide (added as Na_2_S) to achieve ∼100 µM. The average and standard deviation of triplicate measurements is shown.

Growth curves for the two *Stygiolobus* strains isolated from the surface of CP (CP85–0 m) and RP (RP85–0 m) are shown in Fig. 2, while data for the *Stygiolobus* strains isolated from depth at CP (CP85–9 m and CP85–21 m) and the Sulfolobales strain isolated from RB (RB – 0 m) are shown in Figs. S4 and S5, respectively. For both CP85–0 m and RP85–0 m abiotic controls, the sulfide that was added at the start of the experiment quickly equilibrated with the headspace, with 40 µM ± 3.2 µM and 36 µM ± 1.3 µM remaining after 5 h of incubation, respectively (Fig. 2A and D). The rate of sulfide equilibration with the gas phase in these reactors was similar to that observed for the initial abiotic experiment controls lacking O_2_ (Fig. 1B). However, for both strain CP85–0 m and RP85–0 m reactors that contained cells, only 9 µM ± 0.4 µM and 9 µM ± 3.0 µM aqueous sulfide remained, respectively, after 5 h incubation (Fig. 2A and D). Sulfide (as Na_2_S) was again added to CP85–0 m and RP95–0 m biotic reactors to achieve final concentrations of ∼100 µM at 6 and 12 h time intervals. In both biotic reactors, aqueous sulfide was below detection limits (<1 µM) within 6 h of each addition, whereas substantial aqueous sulfide remained in abiotic reactors at these time intervals. After 24 h of incubation, the amount of aqueous sulfide in CP85–0 m abiotic reactors did not change whereas the amount of aqueous sulfide in RP85–0 m abiotic reactors continued to decrease.

In both CP85–0 m and RP85–0 m biotic reactors, the oxidation of sulfide was coupled to production of cells, as indicated by an increase in cell concentrations from 1.1 ± 0.04 × 10^6^ cells mL^−1^ to 7.8 ± 0.4 × 10^6^ cells mL^−1^ and an increase from 8.5 ± 0.3 × 10^5^ to 4.0 ± 0.07 × 10^6^ cells mL^−1^, respectively (Fig. 2B and E). The production of cells and the oxidation of sulfide in the biotic reactors were also accompanied by the production of sulfate (SO_4_^2−^) (Fig. 2C and F). The initial SO_4_^2−^ concentration in CP85–0 m cultures (derived from the 20% vol./vol. of CP hot spring water added) was 422 ± 9 µM and this increased to 620 ± 32 µM after 36 h, yielding a total of 197 µM SO_4_^2−^ produced. This accounted for ∼70% of the sulfide (total of 280 µM H_2_S) that was added to the CP85–0 m reactors during the course of incubation. The initial SO_4_^2−^ concentration in RP85–0 m cultures (derived from the 20% vol./vol. of RP hot spring water added) was 310 ± 9 µM, which decreased to 233 ± 13 µM after 5 h (Fig. 2F), possibly due to assimilation by cells. After 24 h, SO_4_^2−^ increased to 377 µM ± 53 µM, yielding a total of 144 µM SO_4_^2−^ produced. This accounted for ∼51% of the added sulfide (total of 280 µM H_2_S). SO_4_^2−^ production was not detected in abiotic reactors (Fig. 2F). The two *Stygiolobus* strains obtained from depth in CP (CP85–9 m and CP85–21 m) and the Sulfolobales strain isolated from RB (RB85–0 m) exhibited similar cell production, sulfide oxidation kinetics, and SO_4_^2−^ production kinetics when compared to the surface strain from CP (Figs. S4 and S5).

### Comparison of H_2_S- vs. S^0^-dependent growth

**Fig. 3.**
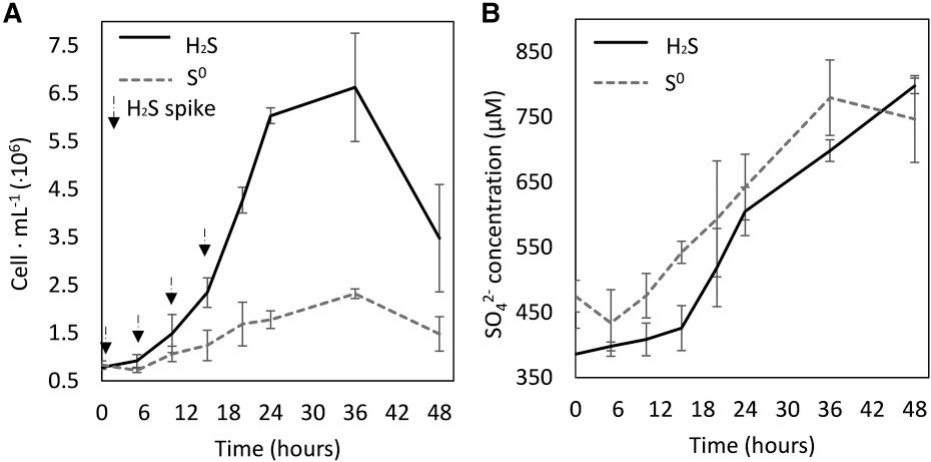
Production of cells (A) and sulfate (SO_4_^2−^; B) in cultures of *Stygiolobus* strain CP85–0 m when grown with sulfide (black solid lines) or elemental sulfur (S^0^, grey dashed lines) as the electron donor. Oxygen (1.5% headspace vol./vol.) was the electron acceptor and carbon dioxide (92% vol./vol.) was the carbon source. Cultures were incubated on a shaker (50 rotations per min) at 80°C. Experiments were conducted in base salts medium with a pH of 2.6, with black arrows indicating the additions of Na_2_S to achieve ∼100 µM in the sulfide growth condition only. Note that the base salts medium, which contains 20% filter-sterilized water from Cinder Pool, had ∼400 µM SO_4_^2−^ at the start of the experiment. The average and standard deviation of triplicate measurements is shown.

The growth kinetics of strain CP85–0 m was examined in sulfide oxidation and S^0^ oxidation conditions. Cultures were grown with the same amount of electron donor (∼450 µM of either Na_2_S or S^0^) and electron acceptor (1.5% O_2_ vol./vol.) with shaking (50 rotations per minute) when incubated at 80°C (Fig. 3). The density of cells in sulfide-grown CP85–0 m cultures increased from 7.8 ± 0.4 × 10^5^ cells mL^−1^ to 6.6 ± 1.1 × 10^6^ cells mL^−1^ during the incubation period whereas the density of S^0^-grown cultures increased from 8.3 ± 0.8 × 10^5^ cells mL^−1^ to 2.3 ± 0.1 × 10^6^ cells mL^−1^, representing roughly 3-fold less growth than the former condition (Fig. 3A). The production of SO_4_^2−^ also differed among growth conditions, with a total of 411 µM SO_4_^2−^ produced in sulfide-grown CP85–0 m cultures (accounting for ∼91% of added H_2_S) whereas only 305 µM SO_4_^2−^ was produced in S^0^-grown CP85–0 m cultures (accounting for ∼68% of added S^0^). The growth efficiency for sulfide-grown cultures was 0.37 cells/picomol SO_4_^2−^ produced and for S^0^-grown cells was 0.09 cells/picomol SO_4_^2−^ produced. The calculated Gibbs energy ((ΔG) at 80°C and 1 atm; Supplemental Methods) for aerobic sulfide-oxidation was −726 kJ mol^−1^ and that for aerobic S^0^-oxidation was −513 kJ mol^1^.

The toxicity of sulfide (added as Na_2_S) to *Stygiolobus* CP85–0 m was also examined (Supplemental Methods, Fig. S6). The number of cells produced when provided with 500 µM sulfide (2.1 ± 0.7 × 10^6^ cells mL^−1^) was similar to when 100 µM sulfide was provided (2.0 ± 0.2 × 10^6^ cells mL^−1^) over the first 24 h of incubation. However, production of cells leveled off in the control condition after 24 h incubation but continued to increase over 72 h in the 500 µM condition (6.5 ± 1.0 × 10^6^ cells mL^−1^), likely reflecting electron donor limitation in the former condition. Cultures of CP85–0 m provided with 1 mM sulfide at the start of the experiment exhibited a lag of 24 h before cell production commenced, ultimately reaching 3.2 ± 1.1 × 10^4^ cells mL^−1^. Like cultures provided with 500 µM sulfide, those provided with 1 mM sulfide reached their highest densities following 72 h incubation (8.2 ± 0.5 × 10^6^ cells mL^−1^). Cultures provided with 15 mM sulfide did not grow, indicating sulfide becomes toxic to CP85–0 m at a concentration between 1 and 15 mM. In abiotic reactors amended with one mM sulfide, and in abiotic and biotic reactors amended with 15 mM H_2_S, a white flocculant precipitate started to form after 0.5 h incubation. The amount of precipitate increased in quantity with incubation time and turned yellowish by the end of the experiment (96 h). The precipitate was identified as S^0^ via XRD (Fig. S7).

### Relevance of sulfide oxidation in Yellowstone hot springs

**Fig. 4.**
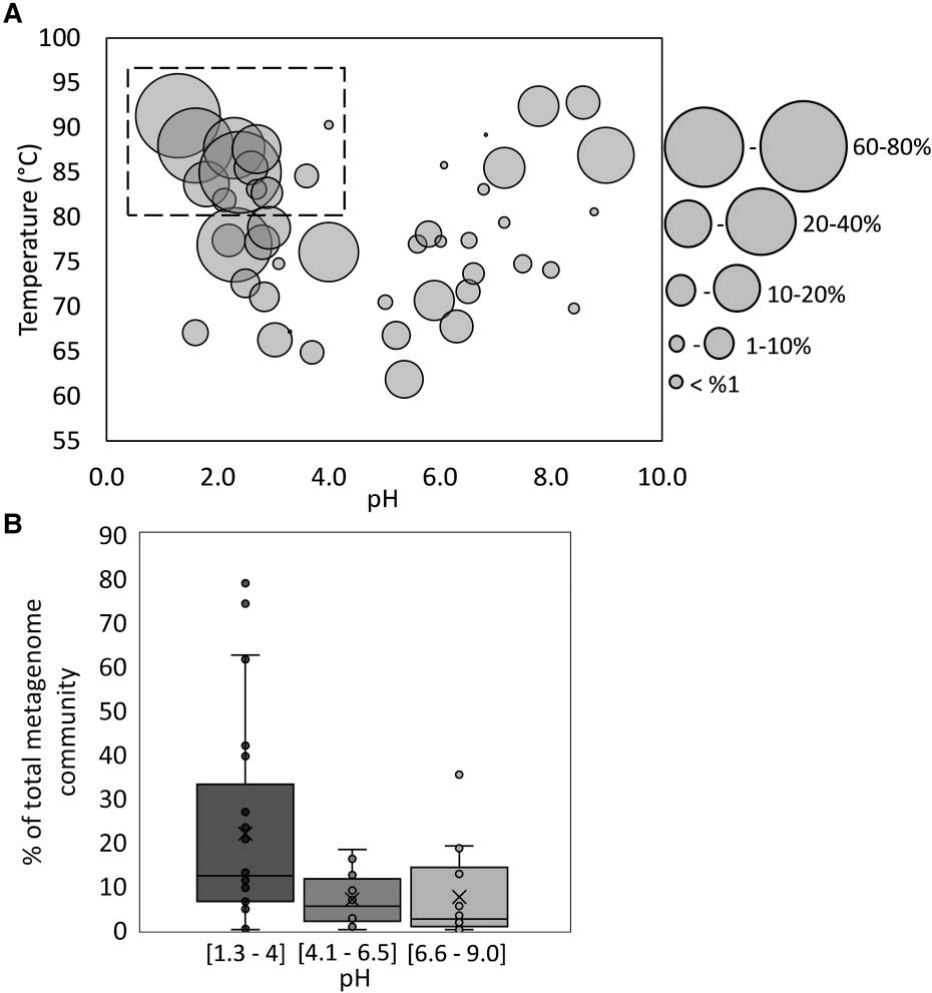
The distribution and relative abundance of sulfide quinone oxidoreductase (Sqr) homologs among metagenome assembled genomes (MAGs) recovered from 51 non-photosynthetic sediment communities from hot springs in Yellowstone National Park. The metagenomes are from hot springs spanning a range of pH (1.3 to 9.0) and temperature (60–93°C). A) Each bubble represents MAGs from one metagenome, and the size represents the sum of the relative abundances of the MAGs that encode a Sqr homolog. Of the 51 metagenomes, four lacked MAGs that did not encode homologs of Sqr and these are not depicted. The box highlights the range of pH and temperature of springs where members of the order Sulfolobales predominate microbial communities (13). B) Boxplots of the relative abundance of Sqr homologs, with the interquartile ranges of distributions denoted by the grey boxes and medians shown as black lines in the center of the boxes. Whiskers show the full ranges of the distributions. The metagenomes were grouped according to pH realms that include acidic hot springs (1.3–4.0), moderately acidic hot springs (4.1–6.5), and neutral to alkaline hot springs (6.6–9.0).

The presence of Sqr homologs was investigated using our in-house compilation of MAGs from 51 hot spring sediment community metagenomes that were recovered across a wide range of pH (1.3–9.0) and temperature (60–93°C) conditions (Fig. 4A). Of the 51 metagenomes examined, MAGs from a metagenome from a low-pH spring (pH 2.9, 81.9°C), MAGs from a metagenome from a mid-pH spring (pH 6.2, 83.2°C), and MAGs from two metagenomes from neutral to alkaline pH springs (pH 7.1, 86.0°C; pH 8.7, 77.2°C) lacked homologs of Sqr. The distribution of Sqr among MAGs from the remaining 47 hot springs was uneven, with those from low pH springs (pH 1.3–4.0; *n* = 24 metagenomes) having significantly (one-way ANOVA 0.019; *P* < 0.05) more Sqr homologs on average than those from mid pH springs (pH 4.1–6.5; *n* = 12 metagenomes) and neutral to alkaline pH springs (pH 6.6–9.0; *n* = 14 metagenomes) (Fig. 4B). The distribution of Sqr homologs among MAGs differed among the low pH hot springs that they came from, with up to 79% of the total metagenome community (as the sum of MAG relative abundance) encoding homologs in one hot spring (pH 1.6, T 88°C). Communities where >60% of the MAGs encoded Sqr homologs were only from hot springs with pH <2.5. In communities from mid-pH hot springs, the highest percentage of MAGs encoding Sqr homologs was 18% whereas all communities from neutral to alkaline hot springs had <20% of the total MAGs, with the exception of one hot spring (pH 9.0, T 87°C) that had up to 35% of the MAGs encoding Sqr homologs. This same spring had 30 µM (1 mg L^−1^) of aqueous sulfide at the time of sampling.

The concentration of aqueous sulfide across 73 YNP hot springs (pH 1.3–9.0; 60–95°C) (Fig. S8) shows that it does not differ as a function of pH (Fig. S8A). However, when SO_4_^2−^ is included in the distribution of total sulfur (sulfide + SO_4_^2−^) in YNP hot springs (S^0^ is not easily measured nor is it often reported) (Fig. S8B), the relationship with pH is apparent with acidic springs being enriched in sulfur. This is consistent with sulfuric acid buffering of low pH (pH < 4) hot springs (7).

### Chemoautotrophic primary production

**Fig. 5.**
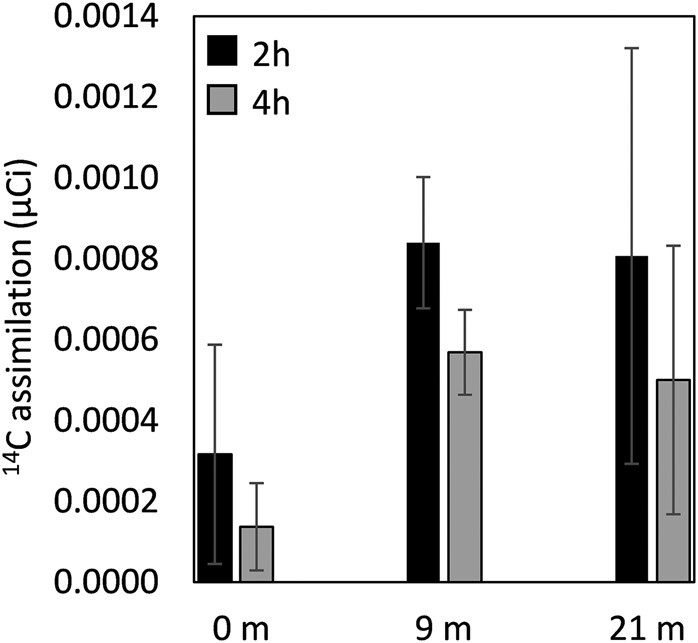
Assimilation of ^14^C-labeled bicarbonate as a proxy of chemosynthetic primary production along a depth profile in Cinder Pool. Quantified activity that is attributable to cells (biotic minus abiotic controls) is plotted as the average and standard deviation of triplicate assays. Black bars depict 2 h of incubation while grey bars depict 4 h of incubation. Data are not normalized to total dissolved inorganic carbon (DIC) uptake to a lack of accurate measurement of native DIC in waters due to extensive degassing from samples pumped from depth.

The isolation of autotrophic *Stygiolobus* strains from the depth at CP prompted experiments to evaluate whether they or other autotrophic members of the community might be active (Supplemental Methods). Chemoautotrophic primary production assays (i.e. dark CO_2_-fixation) were conducted on waters collected from 0, 9, and 21 m depths in CP using ^14^C-labeled bicarbonate. The amount of radioactivity (µCi) fixed into biomass was quantified at 2 and 4 h of incubation at 85°C for abiotic (autoclave sterilized) and biotic assays, and the data shown is that of chemoautotrophic CO_2_-fixation (biotic minus abiotic values) (Fig. 5). The amount of ^14^C-labeled bicarbonate fixed into biomass was higher at 2 h than at 4 h for each of the samples collected from depth, suggesting electron donor/acceptor limitation and/or cell lysis occurred during the course of the assay. On average, the surface samples (0 m) had an activity of 3.1 ± 2.7 × 10^−4^ µCi at 2 h of incubation that decreased to 1.3 ± 1.1 × 10^−4^ µCi at 4 h. The 9 m samples had an activity of 8.9 ± 1.6 × 10^−4^ µCi at 2 h of incubation that decreased to 5.7 ± 1.0 µCi by 4 h. The 21 m samples had an activity 8.0 ± 5.1 × 10^−4^ µCi at 2 h of incubation and decreased to 5.0 ± 3.3 × 10^−4^ µCi at 4 h.

## Discussion

Current models for the generation of acidic hot springs begin by invoking abiotic oxidation of sulfide at high temperature by O_2_, resulting in the formation and accumulation of S^0^ as the stable end-product at low temperatures of <100°C (Eqs. 1–5) through a series of reactions that do not generate net acidity (4, 7, 8, 10, 11). Rather, it is thought that microbially-mediated aerobic oxidation of S^0^ to SO_4_^2−^ and H^+^ (Eq. 6) by members of the archaeal order Sulfolobales drives the acidification of hot springs. However, experiments conducted herein suggest that the rate of high temperature aerobic abiotic oxidation of sulfide at acidic pH is substantially slower than at circumneutral pH and is not instantaneous under either condition, even at atmospheric concentrations of O_2_. The pH dependence of the oxidation kinetics is likely attributable to the pH-dependent protonation of sulfide (47) that protects it from oxidation (Fig. 1), as previously suggested (59). Under suboxic conditions (i.e. 1% O_2_ vol./vol.), which are likely to prevail deeper in springs or in spring sediments, abiotic oxidation of sulfide proceeded even slower at circumneutral pH (pH 7.0) and was negligible at acidic pH (pH 3.0). Together, these observations indicated the presence of a kinetic barrier that allowed low concentrations of sulfide and O_2_ to coexist in acidic and neutral waters at high temperatures. Thus, the possibility exists that microorganisms inhabiting such environments can contribute to, and possibly accelerate, aerobic sulfide oxidation.

Prior studies have suggested that members of the Sulfolobales can oxidize sulfide. For example, a previous report claimed that *A. sulfidivorans* and *A. brierleyi* can grow by oxidizing sulfide with Fe(III) as oxidant (added as Fe_2_(SO_4_)_3_) (39). However, Fe(III) spontaneously reacts with sulfide, forming S^0^ and Fe(II) at acidic pH (60, 61). Further, Fe(II) and S^0^ are a suitable redox pair for many members of the Sulfolobales (19, 61). In the present study, this reaction was shown to occur nearly spontaneously in abiotic reactors containing sulfide and Fe(III) ions (Video S1). S^0^ and Fe(II) were identified as products of the reaction. This indicates that the addition of sulfide by Plumb et al. (2017) to reactors containing Fe(III) ions drove the formation of Fe(II) and S^0^. Thus, *A. sulfidivorans* and *A. brierleyi* were most likely growing via Fe(II) oxidation coupled with S^0^ reduction, rather than via sulfide oxidation coupled with Fe(III) reduction as reported (39).

The lack of characterized thermoacidophiles with the demonstrated ability to catalyze aerobic sulfide oxidation, combined with the demonstration that sulfide and O_2_ can coexist in acidic hot springs and thermodynamic calculations that reveal available free energy from this redox couple (62), motivated new enrichment experiments to isolate strains capable of accelerating aerobic sulfide oxidation. Here, we report five new Sulfolobales strains from YNP hot springs (*Stygiolobus* CP85–0 m, *Stygiolobus* CP85–9 m, *Stygiolobu*s CP85–21 m, *Stygiolobus* RP85–0 m, and Sulfolobales RB85–0 m) that accelerate the oxidation of sulfide under suboxic conditions (Figs. 2, S4, and S5). Each of these strains oxidized ∼300 µM of sulfide over a period of 15 h, bringing the aqueous sulfide concentration to below detection limits (<2 µM). In contrast, sulfide was still quantifiable in abiotic reactors following 36 h of incubation. All strains continued to produce cells between 15 and 24 h incubation after all aqueous sulfide had been consumed, concomitant with the production of SO_4_^2−^ (Figs. 2, S4, and S5). This suggests that the cells continue to oxidize sulfur compounds of intermediate oxidation state (e.g. S_2_O_3_^−^, SO_3_^2−^) once the provided sulfide was consumed. However, these compounds were not detected in spent medium, which is likely due to (i) their localization in the cytoplasm of cells and/or (ii) their inherent instability in oxic and acidic conditions (7).

All four isolated *Stygiolobus* MAGs (CP85–0, 9, and 21 m; RP85–0 m) encoded similar suites of proteins allowing for dissimilatory sulfur metabolism (Fig. S2) including Sqr (sulfide oxidation), Sor and Hdr complex (S^0^ oxidation), and Cox (O_2_-reduction), whereas the isolated Sulfolobales MAG from RB (RB85–0 m) encoded Sqr, the Hdr complex, and Cox (no Sor, a finding that was consistent with the other MAGs of this uncharacterized clade in YNP). Previously, *S. azoricus*, the only species of the genus, was described to be an obligate anaerobe (21) even though its genome encoded Cox. This finding was a departure for the Sulfolobales order, where all characterized members are either obligate or facultative aerobes (13). Recently, a new species, *Stygiolobus caldivivus,* was described and shown to be a facultative aerobe (58), restoring agreement with what it's known of members of the Sulfolobales order as well as adding support to our findings that *Stygiolobus* sp. can utilize O_2_ as an electron acceptor while oxidizing sulfide via Sqr.

Sqr is thought to create linear chains of sulfur as polysulfides (^−^S-S_n_-S^−^) in the cytoplasm (26) that are then disproportionated by sulfur oxygenase:reductase (Sor) that generates additional sulfide, HSO_3_^−^, and ultimately, S_2_O_3_^2−^ (19, 23, 25, 50). HSO_3_^−^ and S_2_O_3_^2−^ react in downstream processes through sulfite:acceptor oxidoreductase (Suox) and thiosulfate:quinone oxidoreductase (Tqo), respectively, and are linked to energy conservation and sulfur trafficking (e.g. through tetrathionate hydrolase [TetH] and the Hdr complex), ultimately generating SO_4_^2−^, H^+^, and ATP (16, 19, 27, 28). However, the sulfide generated from Sor is then re-oxidized by Sqr and the ^−^S-S_n_-S^−^ is again processed by Sor. Thus, a delay in the conservation of energy that can be used to drive biomass production is expected during growth on sulfide until most of the sulfur atoms have travelled through the “energetic spiral” of ∼10 steps following Sqr activity. This is consistent with data shown here (Figs. 2B and E and S4C), where cells experienced a lag phase (∼5–12 h) during the first hours of sulfide-dependent growth. While the Sulfolobales MAG from RB (as well as the whole clade that it belongs to) did not encode homologs of Sor, it encoded homologs of the Hdr complex that has been proposed to be essential for acidophilic and neutrophilic bacteria, and acidophilic archaea (i.e. Sulfolobales) to grow via inorganic sulfur compound oxidation (29–32). Although the exact mechanism of sulfur oxidation via this pathway remains to be elucidated, the Hdr complex is universally conversed in Sulfolobales (Fig. S2) (16, 19, 22), suggesting it is essential and a potential alternative mechanism for intracellular sulfur to be processed after sulfide is oxidized by Sqr. Altogether, these results suggest that the novel Sulfolobales strains isolated in this study and other Sulfolobales in general that encode Sqr, Sor, and/or Hdr can accelerate aerobic sulfide oxidation and conserve energy from this reaction for use in biomass production.

The pKa of sulfide at 80°C is ∼6.4 (47), suggesting that the majority of the sulfide added to reactors in the present study is in the uncharged form, H_2_S, and could freely diffuse across the membrane. H_2_S can be toxic to microorganisms (63–66) due to it interfering with components of electron transport chains and because it can deprotonate once inside the cell (more circumneutral pH) and acidify the cytoplasm (66–68). A previous study by Morales et al. (2011) showed that cultures of *Sulfolobus metallicus* (now called *Sulfuracidifex metallicus*) were tolerant of and able to grow (albeit poorly) on sulfide when provided as a gas (∼1,200 mg L^−1^) in sealed reactors containing 21% O_2_ (∼250 mg L^−1^). However, in abiotic reactors from the same study, ∼300 mg L^−1^ of H_2_S was consumed after 24 h incubation, consistent with abiotic oxidation by O_2_ at near stoichiometric quantities (40). Further, a more recent study (41) showed S­^0^ production in aerobic cultures of *S. metallicus* grown with gaseous H_2_S (1,000 mg L^−1^) over an incubation period of 250 h, with S­^0^ being generated within the first hours of the experiment. Further, tetrathionate was also generated early during the incubation, indicating that both are likely produced as intermediates and were likely available to support growth of this strain (41). Given the poor solubility of H_2_S at pH 2.5 (as shown herein through equilibration experiments), it is possible that intermediate S species supported growth in these experiments, rather than H_2_S. Indeed, experiments conducted herein also show that sulfide at high concentrations reacted with O_2_ to yield S^0^ (Fig. S7). Further, sulfide at a concentration of 1 mM was toxic to *Stygiolobus* CP85–0 m (Fig. S6). Importantly, sulfide concentrations in YNP surface hot spring waters rarely exceed 100 µM (69–72), and reports of concentrations in the mM range found to be toxic to *Stygiolobus* CP85–0 m are even more rare (e.g. Boulder Spring (73)).

We examined whether *Stygiolobus* CP85–0 m could grow with S^0^ and, if so, whether it grew better with S^0^ or sulfide. The strain was grown aerobically (1.5% O_2_ vol./vol.) with ∼450 µM of either H_2_S or S^0^. An important caveat however, is that unlike sulfide, S^0^ is essentially insoluble (478 nM at 80°C) (74). After incubation for 48 h, *Stygiolobus* CP85–0 m cells growing on sulfide (Fig. 2A) achieved a much higher cell density and produced more SO_4_^2−^ (Fig. 2B) than *Stygiolobus* CP85–0 m cells growing on S^0^, revealing that sulfide-grown cells were 4× more efficient in conserving energy and coupling it to growth than S^0^-grown cells. The available free Gibbs energy (ΔG) for both reactions was calculated at a temperature of 80°C and at atmospheric pressure (1 atm). Aerobic sulfide oxidation had a ΔG of −726 kJ mol^−1^ while aerobic S^0^ oxidation had a ΔG of −513 kJ mol^−1^, suggesting that aerobic sulfide oxidation provided 1.4× more energy for the cells than S^0^ oxidation. While it is possible that the rate of sulfide or S^0^ oxidation and the ability to couple this to growth could be influenced by their relative availabilities or solubilities, this alone cannot account for the difference in cell yield. Together these results suggested that *Stygiolobus* CP85–0 m was more efficient at coupling aerobic sulfide oxidation to cell growth than it was at coupling aerobic S^0^ oxidation to growth, possibly pointing to sulfide as the preferred substrate in natural systems. If true, this would represent a stark difference to how Sulfolobales have traditionally been thought to grow in hot spring environments and how they are cultivated in the lab (i.e. via aerobic S^0^ oxidation). Further, these observations provide additional evidence that *Stygiolobus* CP85–0 m, and the other strains isolated in this study, can accelerate the oxidation of sulfide enzymatically and were not simply consuming S^0^ that could have been formed abiotically. Given the ubiquitous distribution of Sqr among members of the Sulfolobales, it is possible that this conclusion can be extended to other Sulfolobales as well.

To begin to probe how relevant aerobic sulfide oxidation via Sqr could be across YNP hot springs, 51 metagenomes that spanned a wide range of pH and temperature were screened for the presence of Sqr homologs. Of the 51 metagenomes, 47 included a MAG that encoded at least one Sqr homolog. The distribution of Sqr homologs (as the sum of the relative abundance of MAGs in a metagenome) was plotted against pH and temperature (Fig. 4). Although the distribution of homologs is quite variable across pH and temperature space, an increased proportion of the community members encode Sqr homologs in acidic pH (<4) and high temperature (>80°C) hot springs. The effect of pH in this distribution was more pronounced when the data from hot spring metagenomes was grouped by broad pH provinces (Fig. 4B). This suggests that the Sulfolobales within YNP, the majority of which remain uncultured (e.g. Acd1) (29, 34, 75), can also possibly accelerate sulfide oxidation.

The majority of Sulfolobales, including all of the new YNP strains, encode the 3HP/4HB pathway of CO_2_ fixation. The isolation of autotrophic strains (*Stygiolobus* CP85–0 m, *Stygiolobus* CP85–9 m, and *Stygiolobus* CP85–21 m were isolated) capable of accelerating sulfide oxidation from various depth intervals in CP, which itself exhibits increasing concentrations of dissolved sulfide with depth (29), motivated experiments to test whether cells residing in the deeper portions of CP might be actively fixing CO_2_ (Fig. 5). Importantly, while MAGs closely affiliated with *Stygiolobus* were not identified at depths up to 15 m in CP (21 m depth was not assayed) in a previous study of CP, they were a minor component (0.03%) of the surface (0 m) planktonic communities (29). Rather, Acd1, a yet to be cultivated member of the Sulfolobales that encodes the 3HP/4HB pathway and Sqr, dominated CP communities regardless of depth. Ex situ ^14^C-labeled bicarbonate activity assays revealed incorporation of ^14^CO_2_ into biomass in planktonic communities collected from all three depth intervals assessed (0, 9, and 21 m) after 2 h incubation. Activities dropped after 4 h incubation, presumably due to nutrient limitation leading to cell lysis. In acidic, high temperature, and sulfur rich hot springs, the nutrient that typically becomes limiting to autotrophs is dissolved O_2_ (76). While total dissolved inorganic carbon (DIC) could not be measured in the samples due to depressurization and degassing during sample collection with a peristaltic pump, it stands to reason that DIC would be more concentrated at depth given the propensity for CO_2_ to volatilize at the surface as waters equilibrate with atmospheric CO_2_. To this end, total measured DIC activity was likely higher at depth, a finding that is consistent with the increased number of cells as a function of increasing depth in CP (29). This likely points to an active in situ community that is fixing CO_2_. Given that autotrophic Sulfolobales that encode Sqr dominate these communities, it seems likely that they may be driving CO_2_ fixation via sulfide oxidation.

## Conclusions

In this study, we investigated abiotic and biotic reactions involving sulfide oxidation at high temperature to test the hypothesis that members of the archaeal order Sulfolobales can accelerate the aerobic oxidation of sulfide and that this, alongside aerobic S^0^-oxidation, contributes to the acidification of hot springs. High temperature abiotic sulfide experiments indicated that the O_2_-dependent oxidation of sulfide at acidic pH was very slow or negligible, with the rate dependent on the concentration of O_2_. This suggested the possibility that Sulfolobales, which universally encode Sqr, could accelerate aerobic sulfide oxidation and potentially couple this to growth. Using sulfide as electron donor and O_2_ as an electron acceptor, five Sulfolobales isolates (four affiliated with *Stygiolobus* and one identifiable only to the Sulfolobaceae family level) were obtained from three springs and from multiple depths below the surface of the spring. All five strains accelerated aerobic sulfide oxidation kinetics while generating biomass and sulfate. Moreover, aerobic growth was better with sulfide when compared to S^0^-oxidation as indicated by a 4× higher growth efficiency, attributable in part to the 1.4× more energy released by aerobic sulfide oxidation than S^0^-oxidation. However, sulfide was toxic at concentrations of >1 mM, which is above the concentration of sulfide typically measured in YNP.

Metagenomic data indicated that Sqr homologs are enriched in acidic hot springs where Sulfolobales predominate. Combined with data presented here suggesting Sulfolobales can accelerate sulfide oxidation, a new model for hot spring acidification is advocated wherein the initial O_2_-dependent oxidation of sulfide is no longer only considered to be an abiotic process. Rather, Sulfolobales accelerate the O_2_-dependent oxidation of sulfide that is used to drive primary production and this results in the production of acid. It is possible that O_2_ limitation limits the biological capacity to oxidize sulfide in some springs or, at certain times in a given spring, it can lead to the generation of intermediate sulfur species (e.g. S_2_O_3_^2−^) that have been measured in acidic springs, and ultimately, metastable S^0^ that can precipitate and accumulate. Sulfolobales-mediated oxidation of S^0^ likely further contributes to the acidification of hot spring waters. Additional research is still needed to uncover whether Sulfolobales prefer S^0^ or sulfide when both substrates are present and the relative contribution of abiotic and biotic processes to sulfide oxidation and spring acidification.

## Supplementary Material

pgae201_Supplementary_Data

## Data Availability

All sequencing data generated by this study are available under NCBI BioProject accession number PRJNA1019763. All geochemical data generated by this study are available in tables and supplemental tables.
