# Supplementary material for: Sulfide oxidation by members of the Sulfolobales
Source: PNAS Nexus. 2024 May 23;3(6):pgae201. doi: 10.1093/pnasnexus/pgae201 (PMC11143483; doi:10.1093/pnasnexus/pgae201)
Supplement: pgae201_Supplementary_Data [file pgae201_supplementary_data.zip › PNASNEXUS-PNASNEXUS-2023-01357R-s01.docx]

**Tables**

Supplementary Table 1. Location and geochemical characteristics of hot spring waters used to isolate H_2_S-oxidizing members of the Sulfolobales. Samples of surface waters (0 m) from ‘Realgar Pool’ and Cinder Pool were collected on August 28^th^, 2020, and samples from depth (9 m and 21 m) at Cinder Pool were collected on June 3^rd^, 2021. Samples of surface waters (0 m) from ‘Red Bubbler’ were collected on June 13^th^, 2023. A more complete geochemical analysis is reported in Supp. Table 1.

| Site | GPS | pH | Temp. (°C) | Cond. (mS) | SO_4_^2-^ (mg/L) | Cl^-^ (mg/L) | S^2-^ (mg/L) | Fe (II) (mg/L) |
| --- | --- | --- | --- | --- | --- | --- | --- | --- |
|  | N W |  |  |  |  |  |  |  |
| ‘Realgar Pool' | 44.73558 110.70705 | 3.9 | 85.8 | 4.44 | 420 | 542 | 0.19 ± 0.04 | 0.43 |
| Cinder Pool, 0 m | 44.43568 110.42351 | 2.6 | 87.8 | 5.88 | 360 | 579 | 0.06 ± 0.01 | 0.25 |
| Cinder Pool, 9 m | 44.43568 110.42351 | 2.6 | 88.2 | 5.49ᵃ | 306ᵃ | NA | 0.16 ± 0.03ᵃ | NA |
| Cinder Pool, 21 m | 44.43568 110.42351 | 2.6 | 91.0 | NA | 350 | NA | NA | NA |
| ‘Red Bubbler' | 44.72650 110.70900 | 3.0 | 90.0 | 2.82 | 220 | 266 | BD | 5.8 |

BD, below detection; NA, not available; ᵃ Measurements from ^29^.
