# Supplementary material for: Sulfide oxidation by members of the Sulfolobales
Source: PNAS Nexus. 2024 May 23;3(6):pgae201. doi: 10.1093/pnasnexus/pgae201 (PMC11143483; doi:10.1093/pnasnexus/pgae201)
Supplement: pgae201_Supplementary_Data [file pgae201_supplementary_data.zip › PNASNEXUS-PNASNEXUS-2023-01357R-s10.docx]

**Supplemental Methods**

Sulfide Oxidation by Members of the Sulfolobales

Maria C. Fernandes-Martins^1^, Daniel R. Colman^1^ and Eric S. Boyd^1,*^

*^1^Department of Microbiology and Cell Biology, Montana State University, Bozeman, Montana*

**X-Ray Powder Diffraction Analysis.** Precipitates that were observed during abiotic reactions and growth of Sulfolobales strains were concentrated via centrifugation (14,000 × *g*, 20 min., 4°C) from triplicate reactors (~100 mL in total). The supernatant was discarded via pipetting and the pellet was air dried overnight. Precipitates were characterized at the Imaging and Chemical Analysis Laboratory (ICAL) at Montana State University using a SCINTAG X-1 system X-ray powder diffraction (XRD) spectrometer (XRD Eigenmann GmbH, Mannheim, Germany).

**Scanning Electron Microscopy (SEM).** Ten mL of the enrichment culture from *Stygiolobus* sp. CP85 – 0m growing on sulfide were harvested during log-phase via centrifugation (14,000 × *g*, 20 min., 4°C) for field emission scanning electron microscope (FE-SEM) analysis. After centrifugation, the supernatant was removed by aspiration and the cell pellet was resuspended in base salt medium containing 2% vol./vol. glutaraldehyde for 2 h at room temperature (~21°C). Fixed cells were collected on an Au-sputtered 0.2 µm black polycarbonate filter and were subjected to dehydration using an ethanol series (25, 50, 70, 85, 95, and 100%). Filtered and dehydrated cells were stored dry at 4°C until imaging in the Imaging and Chemical Analysis Laboratory (ICAL) at Montana State University, as previously described [1].

**Energetics Calculations.** Available Gibbs free energy (ΔG) at the specific pressure and temperature of growth experiments (1 atm; 80°C) at the time of inoculation was calculated using the package CHNOSZ as implemented in the R statistical framework [2]. ΔG calculations can be described by Equation 7 [3]:

ΔG = ΔG° + 2.303RT logQ (Equation 7)

H_2_S (aq) + 2 O_2_ (aq) → HSO_4_^-^ (aq) + H^+^ (Equation 8)

where ΔG° represents standard conditions (J mol ^-1^), R represents the ideal gas constant (~8.314 J mol^-1^ K^-1^), T indicates temperature in Kelvin (K), and Q is the reactant and product activity quotient. Q was calculated using the balanced equation for aerobic S^0^ oxidation (Eq. 6) and for aerobic sulfide oxidation (Eq. 8) at the specific ionic strength of the aqueous solutions.

**Determination of Sulfide Toxicity.** The *Stygiolobus* CP85 – 0m strain was used to probe the concentration where sulfide becomes toxic. Cultivation medium was prepared as described above but was amended to include different starting concentrations of sulfide, added as Na_2_S. This included 100 µM (positive control), 500 µM, 1 mM and 15 mM starting sulfide. All culture vials were amended with citrate buffer at a concentration to sufficiently buffer the medium (pH 2.6) given the amount of Na_2_S that was added to achieve the final sulfide concentration (i.e., the 500 µM sulfide treatment included 600 µM citrate buffer). Cultures were monitored every 24 h to monitor the depletion of sulfide via the methylene blue assay [4] and the production of cells via fluorescent microscopy, as described above.

**Dissolved Inorganic Carbon Assimilation Assays.** Rates of dissolved inorganic carbon assimilation were determined for planktonic-associated communities recovered from a depth profile (0 m, 9 m, 21 m) in ‘Cinder Pool’ (pH 2.6, T 88°C), as previously described [5]. Briefly, acid-washed 24 mL serum bottles were sealed with butyl rubber stoppers and purged with N_2_ that had been passed over heated (>200°C) and H­_2_-reduced copper shavings for 5 min. before autoclaving. In the field, 10 mL of spring water from each of the three depth profiles was added directly to twelve serum vials; the gas phase in the vials was equalized to atmospheric pressure using a sterile needle and syringe. All microcosm vials prepared in the field were immediately placed on ice and in the dark for the 2 hr transport back to the laboratory. In the laboratory, six microcosms from each depth profile were subjected to a single 20 min. autoclave cycle (121°C, 20 psi) for use as abiotic controls. Microcosm vials were brought to room temperature (~21°C) and five μCi of [^14^C] sodium bicarbonate (NaH^14^CO_3_) was added to each vial. Microcosms were placed in a sealed bag (secondary containment) and incubated in the dark near the measured temperature of hot spring waters (*see* Table 1) for two and four hrs. After incubation, microcosms were placed in a second sealed bag (tertiary containment) and stored at -20°C until they were processed, as previously described [5].

**References**

1. Payne, D., R.L. Spietz, and E.S. Boyd, *Reductive dissolution of pyrite by methanogenic archaea.* The ISME Journal, 2021. **15**(12): p. 3498-3507.

2. Boyer, G., *pyCHNOSZ: Python wrapper for the thermodynamic package CHNOSZ* 2023: [**https://github.com/worm-portal/AqEquil**](https://github.com/worm-portal/AqEquil).

3. Amenabar, M.J., et al., *Microbial substrate preference dictated by energy demand rather than supply.* Nature Geoscience, 2017. **10**(8): p. 577-581.

4. Fogo, J.K. and M. Popowsky, *Spectrophotometric Determination of Hydrogen Sulfide.* Analytical Chemistry, 1949. **21**(6): p. 732-734.

5. Fernandes-Martins, M.C., D.R. Colman, and E.S. Boyd, *Relationships between fluid mixing, biodiversity, and chemosynthetic primary productivity in Yellowstone hot springs.* Environ Microbiol, 2023.
