# Supplementary material for: Sulfide oxidation by members of the Sulfolobales
Source: PNAS Nexus. 2024 May 23;3(6):pgae201. doi: 10.1093/pnasnexus/pgae201 (PMC11143483; doi:10.1093/pnasnexus/pgae201)
Supplement: pgae201_Supplementary_Data [file pgae201_supplementary_data.zip › PNASNEXUS-PNASNEXUS-2023-01357R-s11.docx]

**Supplemental Figures**


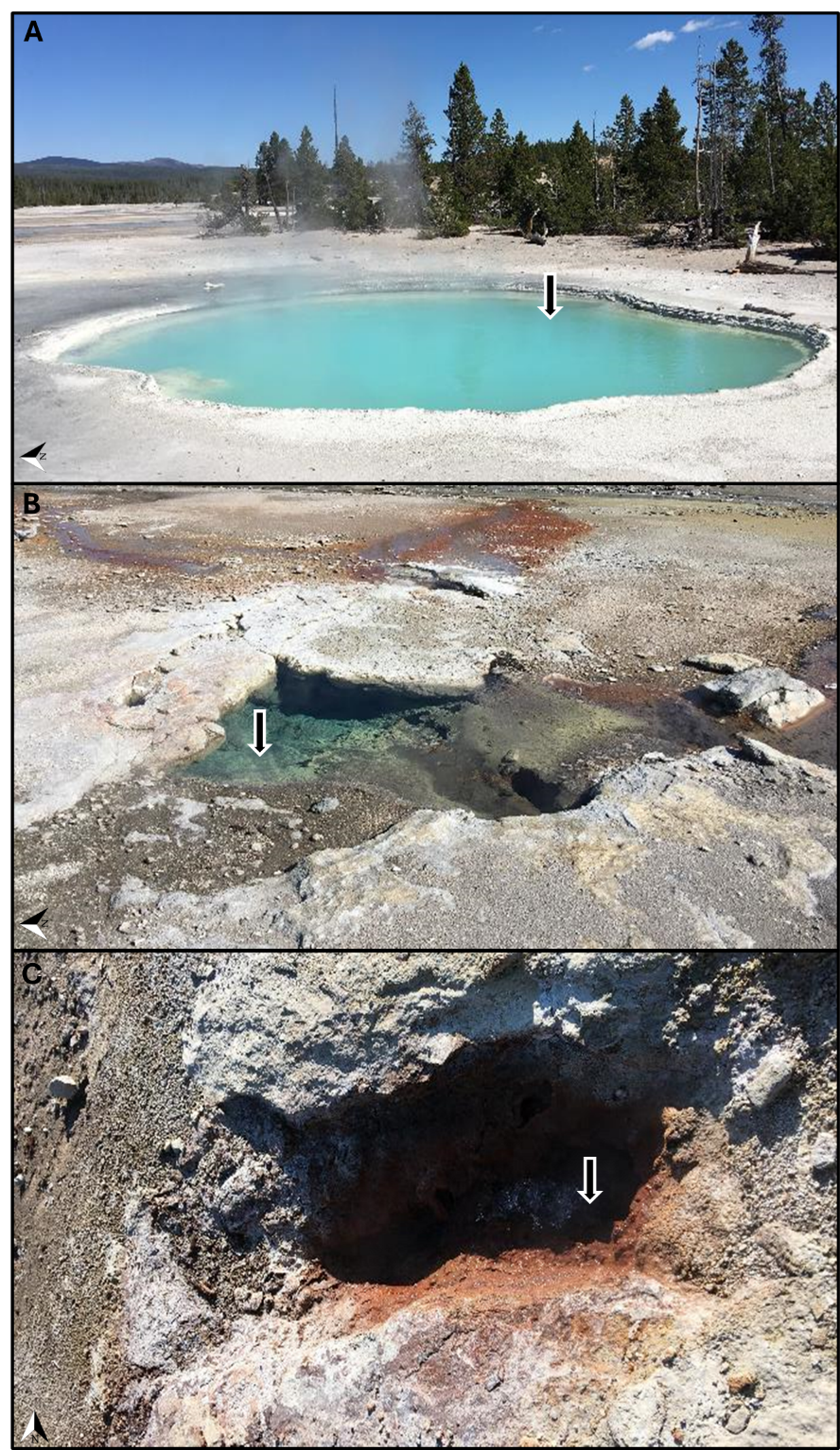


**Supplemental Figure 1. Images of hot springs in Norris Geyser Basin, Yellowstone National Park where samples were collected for cultivation.** The arrows denote the specific location where sediments (or waters) were collected from Cinder Pool (**A**), ‘Realgar Pool’ (**B**), and ‘Red Bubbler’ (**C**). The cardinal north symbol in the bottom left of each panel is shown for reference.


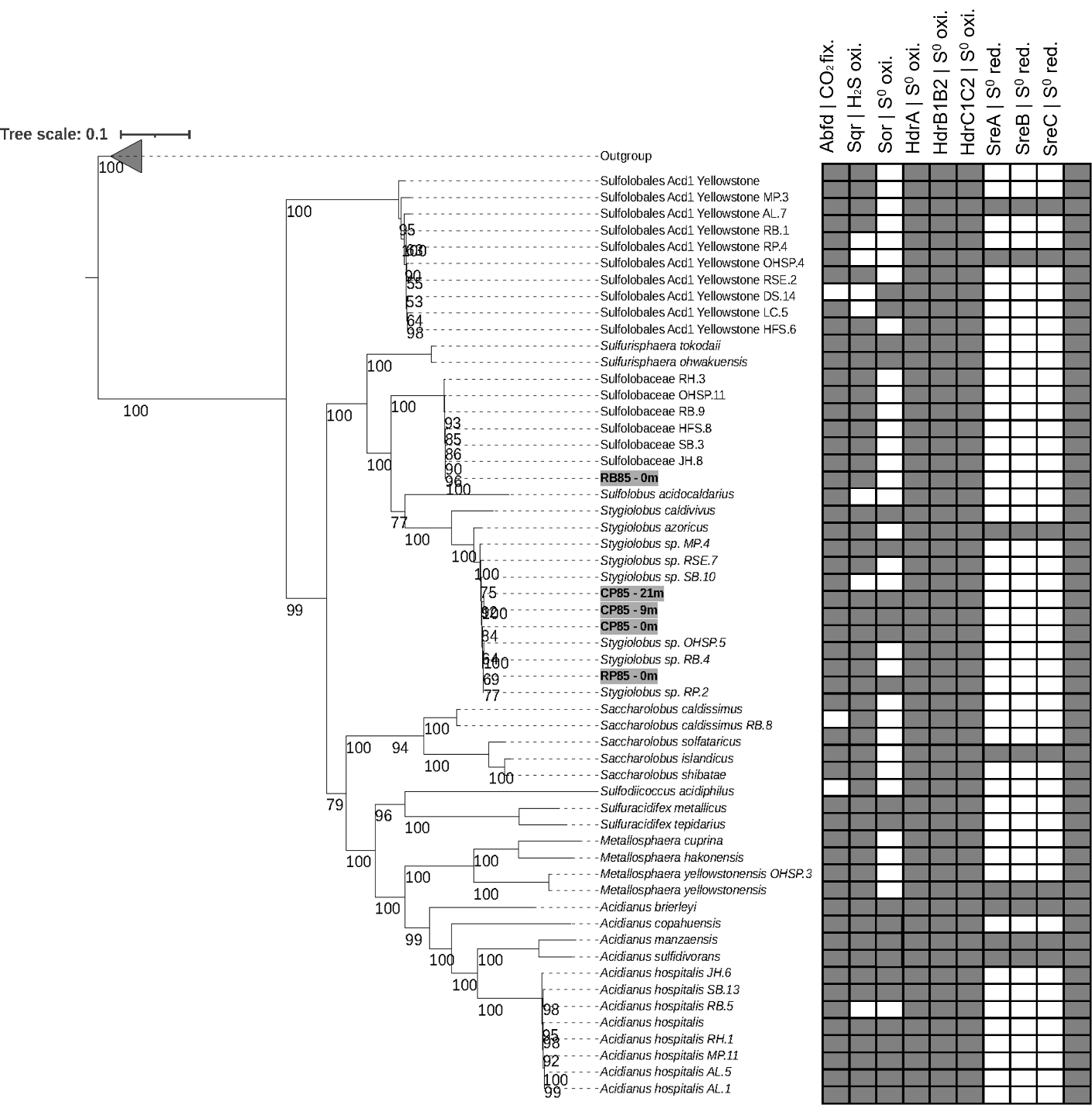


**Supplemental Figure 2. Phylogenomic reconstruction of representative members of the archaeal order Sulfolobales and Sulfolobales isolates recovered in this study (bold-faced and grey-shaded)**. The Maximum-Likelihood phylogeny was constructed using an alignment of marker genes (*n* = 30) and the LG substitution model. Homologs of genes encoding key sulfur-metabolizing enzymes mapped to each metagenome assembled genome or genome (grey shade indicates presence). Abbreviations: Abfd: 4-hydroxybutanoyl-CoA dehydratase; Sqr: sulfide:quinone oxidoreductase; Sor: sulfur oxidoreductase:reductase; HdrAB1B2C1C2: hetereodisulfide reductase; SreABC: sulfur/polysulfide reductase; Cox: cytochrome *c* oxidase subunit I.


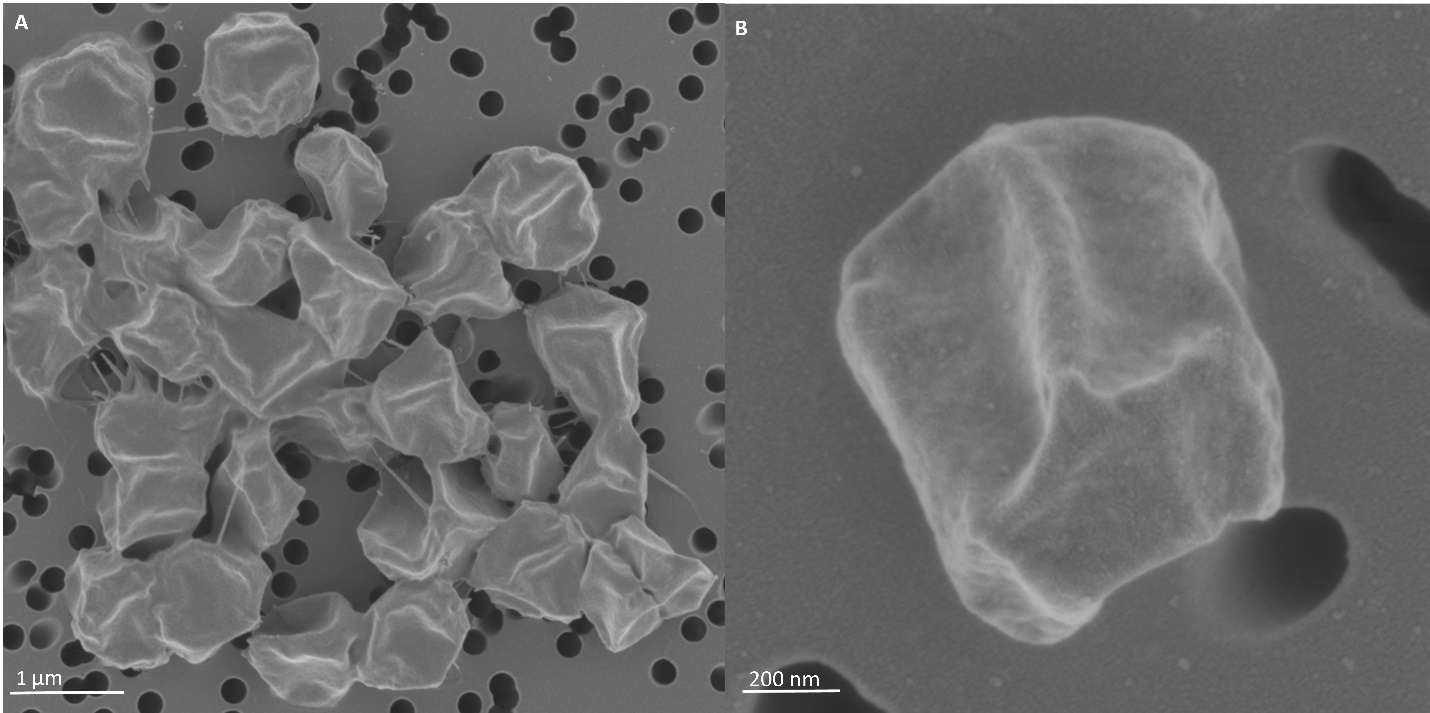


**Supplemental Figure 3. Field emission-scanning electron microscopy images of *Stygiolobus* CP85 – 0m.** Cells were grown with sulfide (added as Na_2_S) as electron donor (total of ~280 µM), oxygen as electron acceptor (1.5% headspace vol./vol.), and carbon dioxide (92% vol./vol.) as carbon source. Cultures were grown in base salts medium with a pH of 2.6 and were incubated at 85°C. An image of a cluster of cells (**A**) and an image of a single cell (**B**) are shown.


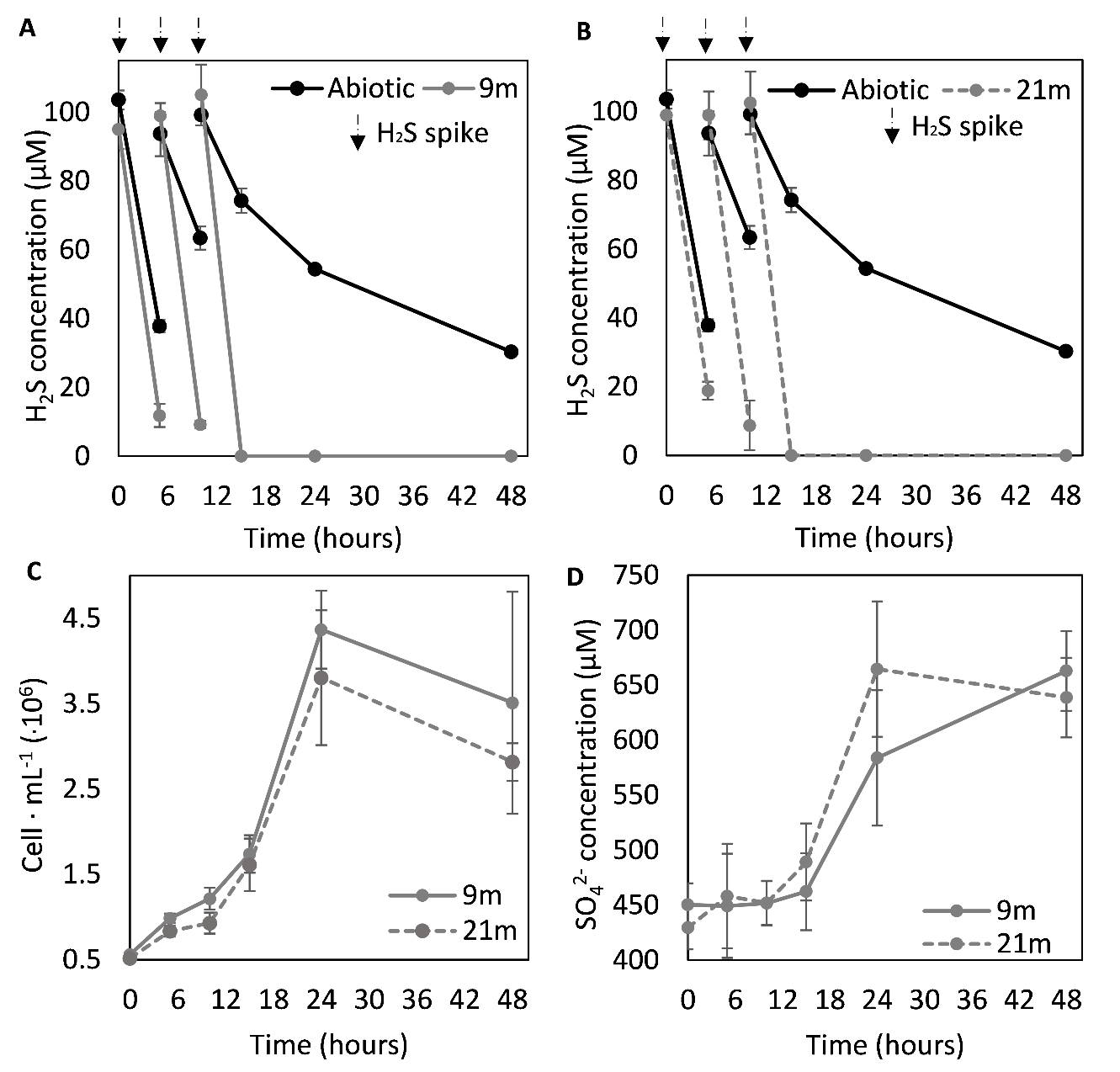


**Supplemental Figure 4. Depletion of aqueous sulfide, production of cells, and production of sulfate (SO_4_^2-^) in cultures of *Stygiolobus* strain CP85 – 9 m (A, C, D) and *Stygiolobus* strain CP85 – 21 m (B, C, D).** Cultures were incubated at 85°C with oxygen (1.5% headspace vol./vol.) as the electron acceptor and carbon dioxide (92% vol./vol.) as the carbon source. Experiments were conducted in base salts medium with a pH of 2.6. Black arrows depict additions of sulfide (as Na_2_S) to achieve ~100 µM. The average and standard deviation of triplicate measurements is shown.


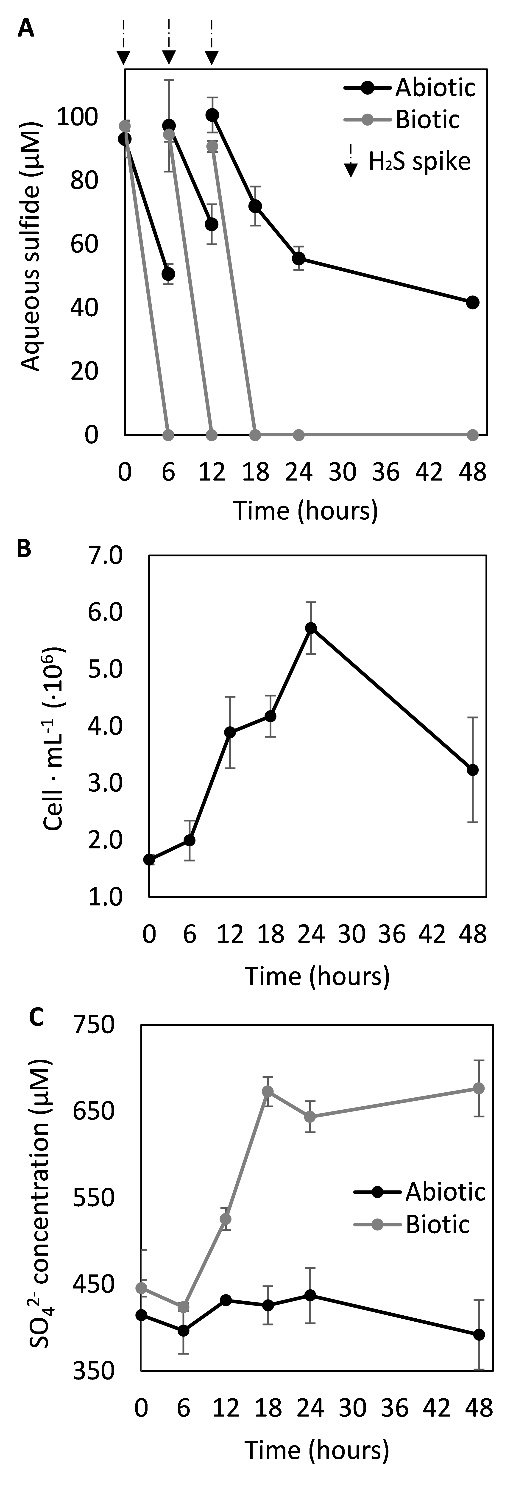


**Supplemental Figure 5. Depletion of total sulfide (A), production of cells (B), and production of sulfate (SO_4_^2-^, C).** Data are shown for cultures of Sulfolobales strain RB85 – 0m when incubated at 85°C**.** Oxygen (1.5% headspace vol./vol.) was the electron acceptor and carbon dioxide (92% vol./vol.) was the carbon source. Experiments were conducted in base salts medium with a pH of 3.0. Black arrows depict additions of Na_2_S to achieve ~100 µM total sulfide. The average and standard deviation of triplicate measurements is shown.


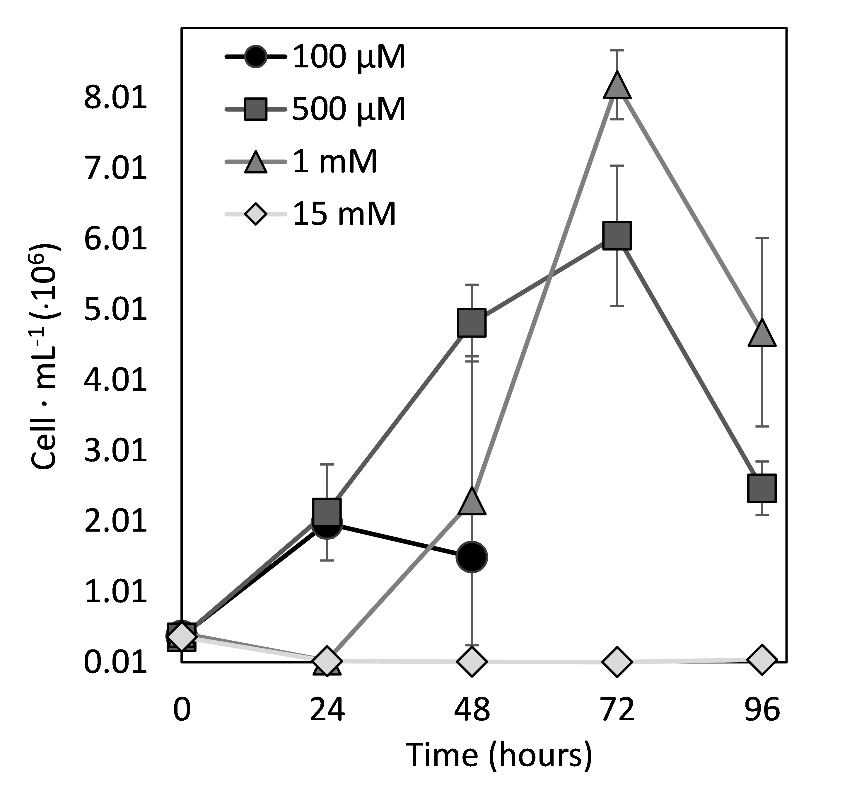


**Supplemental Figure 6. Suppression of cell production in cultures of *Stygiolobus* sp. CP85 – 0 m grown in the presence of increasing concentrations of aqueous sulfide when incubated at 85°C.** Sulfide was provided as the electron donor, oxygen (1.5% headspace vol./vol.) as the electron acceptor, and carbon dioxide (92% vol./vol.) as the carbon source. Experiments were conducted in base salts medium with a pH of 2.6. Sulfide (as Na_2_S) was added at the start of the experiment (time 0 hr) only. Experiments were terminated for each condition after cell production ceased. The average and standard deviation of triplicate measurements is shown.


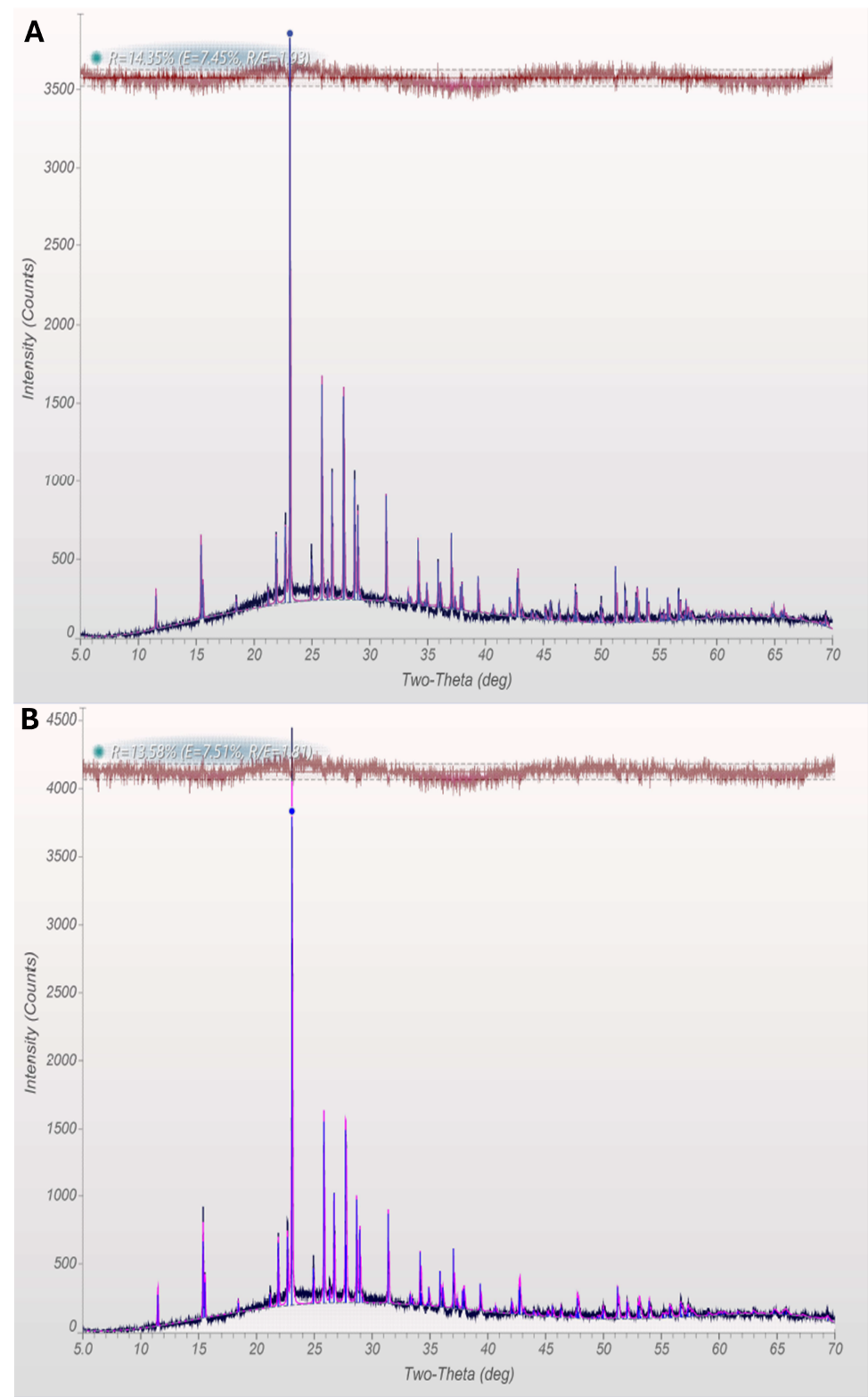


**Supplemental Figure 7. X-ray diffraction (XRD) spectra of precipitates formed in abiotic vials containing ferric iron [Fe(III) added as (Fe_2_SO_4_)_3_] and sulfide (added as Na_2_S) (A), and in abiotic and biotic vials containing 15 mM sulfide (added as Na_2_S) with 1.5% O_2_ (B), at 85°C.** The vials for (A) contained 100% base salt media with a pH of 2.6, whereas vials for (B) contained a 4:1 ratio of base salt media to filtered and autoclaved hot spring water, with a pH of 2.6.


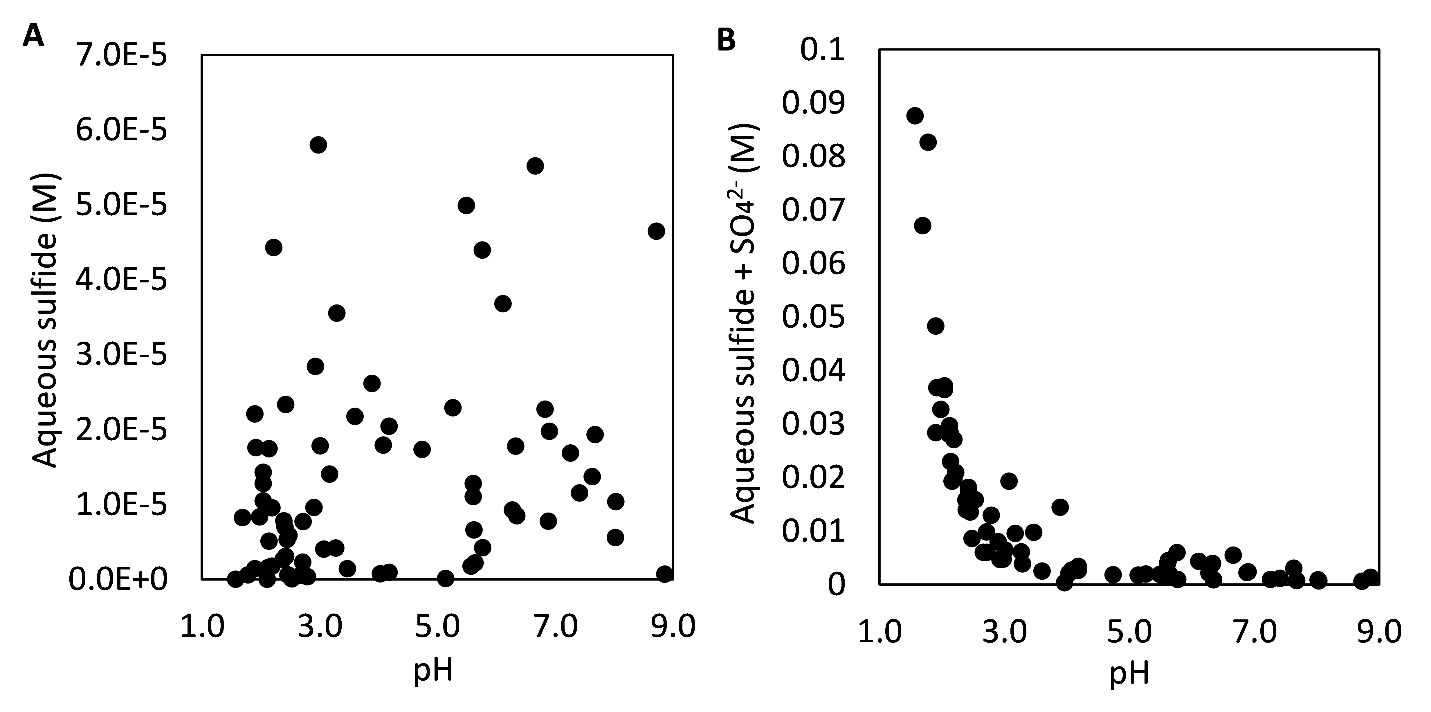


**Supplemental Figure 8. Concentrations (in molar) of aqueous sulfide (A) and total sulfide plus sulfate (SO_4_^2-^) (B) in 73 hot springs in Yellowstone National Park.** Data was obtained from an open file report from the United States Geological Survey ^71^ and span a range of pH (1.3-9.0) and temperature (60-95°C).

**Legend for Supplementary Video 1**

**Supplementary Video 1.** Instantaneous abiotic reaction with 25 mM (Fe_2_SO_4_)_3_ upon addition of 8 mM sulfide (added as Na2S) at pH 2.6 and 80°C.
